# Efficacy of the monocarbonyl curcumin analog C66 in the reduction of diabetes-associated cardiovascular and kidney complications

**DOI:** 10.1186/s10020-022-00559-5

**Published:** 2022-10-31

**Authors:** Mitko Mladenov, Jane Bogdanov, Bogdan Bogdanov, Nikola Hadzi-Petrushev, Andre Kamkin, Radoslav Stojchevski, Dimiter Avtanski

**Affiliations:** 1 Faculty of Natural Sciences and Mathematics, Institute of Biology, Ss. Cyril and Methodius University in Skopje, Skopje, Macedonia; 2 Department of Physiology, Pirogov Russian National Research Medical University, Ostrovityanova Street 1, Moscow, Russia; 3 Faculty of Natural Sciences and Mathematics, Institute of Chemistry, Ss. Cyril and Methodius University in Skopje, Skopje, Macedonia; 4 Friedman Diabetes Institute at Lenox Hill Hospital, Northwell Health, 110 E 59th Street, Suite 8B, Room 837, 10022 New York, NY USA; 5 Feinstein Institutes for Medical Research, Manhasset, NY USA; 6 Donald and Barbara Zucker School of Medicine at Hofstra/Northwell, Hempstead, NY USA

**Keywords:** Curcumin analogs, C66, Diabetes mellitus, Aorta, Heart, Kidney

## Abstract

Curcumin is a polyphenolic compound derived from turmeric that has potential beneficial properties for cardiovascular and renal diseases and is relatively safe and inexpensive. However, the application of curcumin is rather problematic due to its chemical instability and low bioavailability. The experimental results showed improved chemical stability and potent pharmacokinetics of one of its analogs – (2*E*,6*E*)-2,6-bis[(2-trifluoromethyl)benzylidene]cyclohexanone (C66). There are several advantages of C66, like its synthetic accessibility, structural simplicity, improved chemical stability (in vitro and in vivo), presence of two reactive electrophilic centers, and good electron-accepting capacity. Considering these characteristics, we reviewed the literature on the application of C66 in resolving diabetes-associated cardiovascular and renal complications in animal models. We also summarized the mechanisms by which C66 is preventing the release of pro-oxidative and pro-inflammatory molecules in the priming and in activation stage of cardiomyopathy, renal fibrosis, and diabetic nephropathy. The cardiovascular protective effect of C66 against diabetes-induced oxidative damage is Nrf2 mediated but mainly dependent on JNK2. In general, C66 causes inhibition of JNK2, which reduces cardiac inflammation, fibrosis, oxidative stress, and apoptosis in the settings of diabetic cardiomyopathy. C66 exerts a powerful antifibrotic effect by reducing inflammation-related factors (MCP-1, NF-κB, TNF-α, IL-1β, COX-2, and CAV-1) and inducing the expression of anti-inflammatory factors (HO-1 and NEDD4), as well as targeting TGF-β/SMADs, MAPK/ERK, and PPAR-γ pathways in animal models of diabetic nephropathy. Based on the available evidence, C66 is becoming a promising drug candidate for improving cardiovascular and renal health.

## Background

### Curcumin and diabetes mellitus

**Fig. 1:**
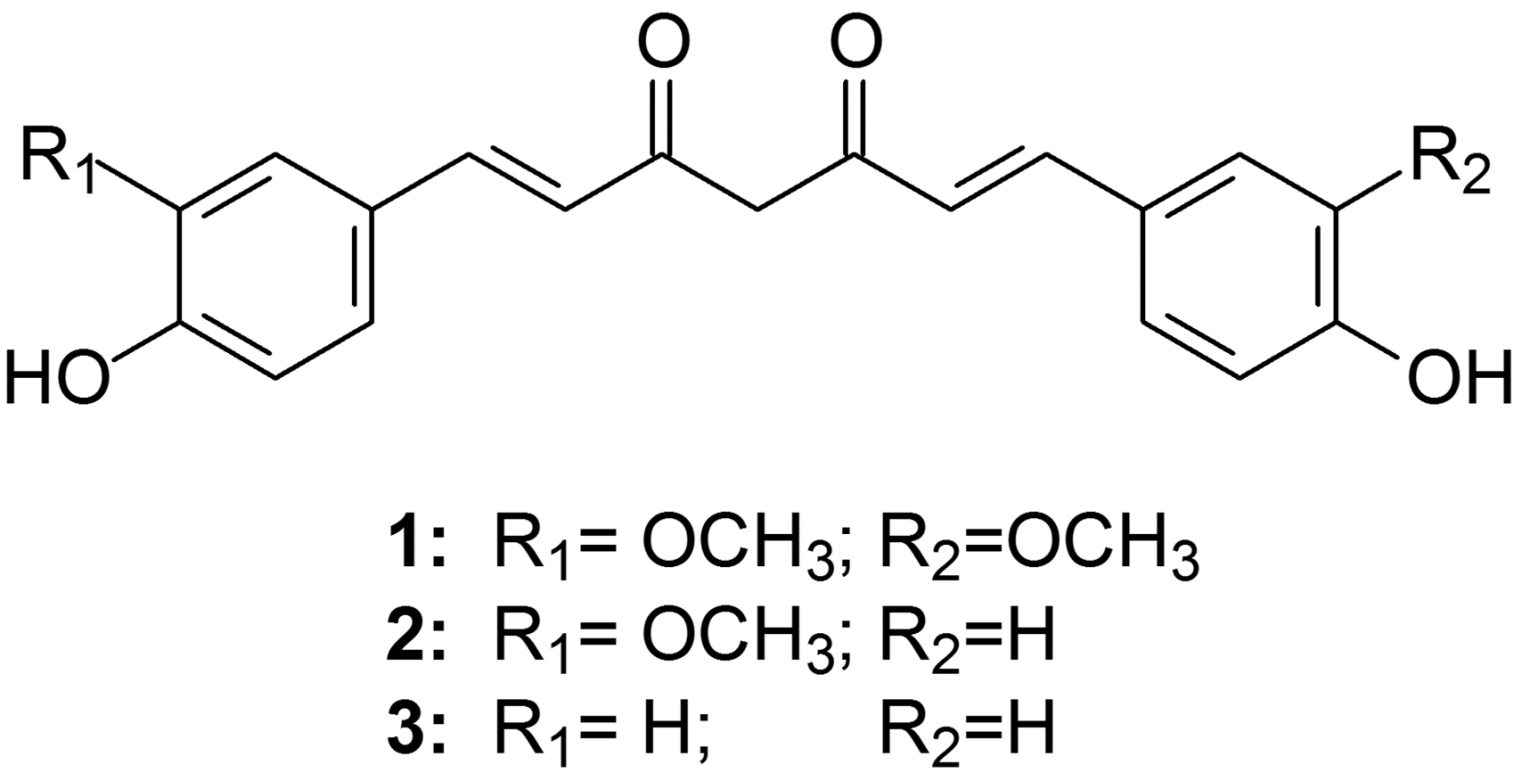
**Chemical structures of curcuminoids. (1)** Curcumin, (1E,6E)-1,7-Bis(4-hydroxy-3-methoxyphenyl)hepta-1,6-diene-3,5-dione; **(2)** Demethoxycurcumin, (1E,6E)-1-(4-Hydroxy-3-methoxyphenyl)-7-(4-hydroxyphenyl)hepta-1,6-diene-3,5-dione; **(3)** Bisdemethoxycurcumin, (1E,6E)-1,7-Bis(4-hydroxyphenyl)hepta-1,6-diene-3,5-dione

Diabetes mellitus is estimated to currently affect almost half a billion people worldwide as this number is expected to reach 700 million by 2045 (Cho et al. 2018). Although conventional treatment regimens remain a priority, natural compounds provide an attractive alternative as supplemental therapy in terms of their pleiotropic actions and low side effects. The biological activities of turmeric (*Curcuma longa*, L.), used for centuries in culinary and traditional medicine for treating conditions of the cardiovascular, pulmonary, digestive, renal, and nervous systems as well as its anti-bacterial and anti-pathogenic properties, are well known (Gupta et al. 2013a, b; Maheshwari et al. 2006). Main bioactive compounds in turmeric are curcuminoids (curcumin (1), demethoxycurcumin (2) and bisdemethoxycurcumin (3), Fig. 1), sesquiterpenoids, and turmerones. Among these compounds, curcumin is the most abundant and active that has been shown to possess anti-inflammatory actions, reduce insulin resistance, decrease glucose and insulin levels, and increase adiponectin release (Anand et al. 2008). Additionally, curcumin reduces the levels of resistin, leptin, IL-6, IL-1β, and TNF-α in patients with type 2 diabetes (Marton et al. 2021; Parsamanesh et al. 2018; Hajavi et al. 2017). The research carried out indicates that curcumin can exert an influence on glucose homeostasis and may help to alleviate the vascular risk in diabetic patients (Pivari et al. 2019). Also, some studies have shown that treatment with curcuminoids improves the lipid profile and increases the overall antioxidant capacity in the blood (Panahi et al. 2017a, b; Altobelli et al. 2021).

As curcumin possesses strong hypoglycemic, hypolipidemic, anti-inflammatory, and antioxidant properties, the treatment of diabetes-related complications is one area where it can be exploited. Curcumin can be used as a supplement alongside the conventional diabetes mellitus therapy, following a thorough testing for its compatibility with the antidiabetic drugs (Rivera-Mancía et al. 2018). However, a caution is required, since curcumin is rather problematic due to its chemical instability, poor aqueous solubility and low bioavailability (Nelson et al. 2017). To overcome these drawbacks, various approaches, such as use of adjuvant or inclusion in drug delivery systems (liposomes or nanoparticles), have been considered. Another strategy for improving curcumin bioavailability is through structural modification of its molecule (Fig. 1).

In many natural products, there is a presence of 2-methoxyphenol scaffold usually substituted at position 4 (eugenol, isoeugenol, vanillin, vanillic acid, coniferyl alcohol, ferulic acid, guaiacol). Compounds that have a methoxy group ortho to the phenolic hydroxyl group are potent antioxidants. The 4-substituted − 2-methoxyphenol moiety is related to the biological activity (antioxidant properties and antimicrobial properties) in compounds. The downfall is that they are prone to oxidation which affects their stability and subsequent use.

### Bioavailability-associated structural modifications of curcumin (monocarbonyl analogs of curcumin)

Even though curcumin is frequently used in research studies, its origin, crystal form and purity prior to use is rarely explicitly provided. Furthermore, the fact that lately curcumin has been classified as PAINS (pan-assay interference compounds), as well as IMPS (invalid metabolic panaceas), must also be taken into account (Nelson et al. 2017). Indeed, only recently, the rich chemistry of curcumin and the role of the auto-oxidation products in its overall activity has been demystified (Gordon et al. 2015a, b; Schneider et al. 2015). Curcumin is photoreactive and it decomposes mainly by solvolysis and oxidative degradation (Nelson et al. 2017). Moreover, it does not satisfy the primary criterion for pharmaceutical applications i.e., it is unstable under physiological conditions (pH 7.4 and 37 °C).

**Fig. 2:**
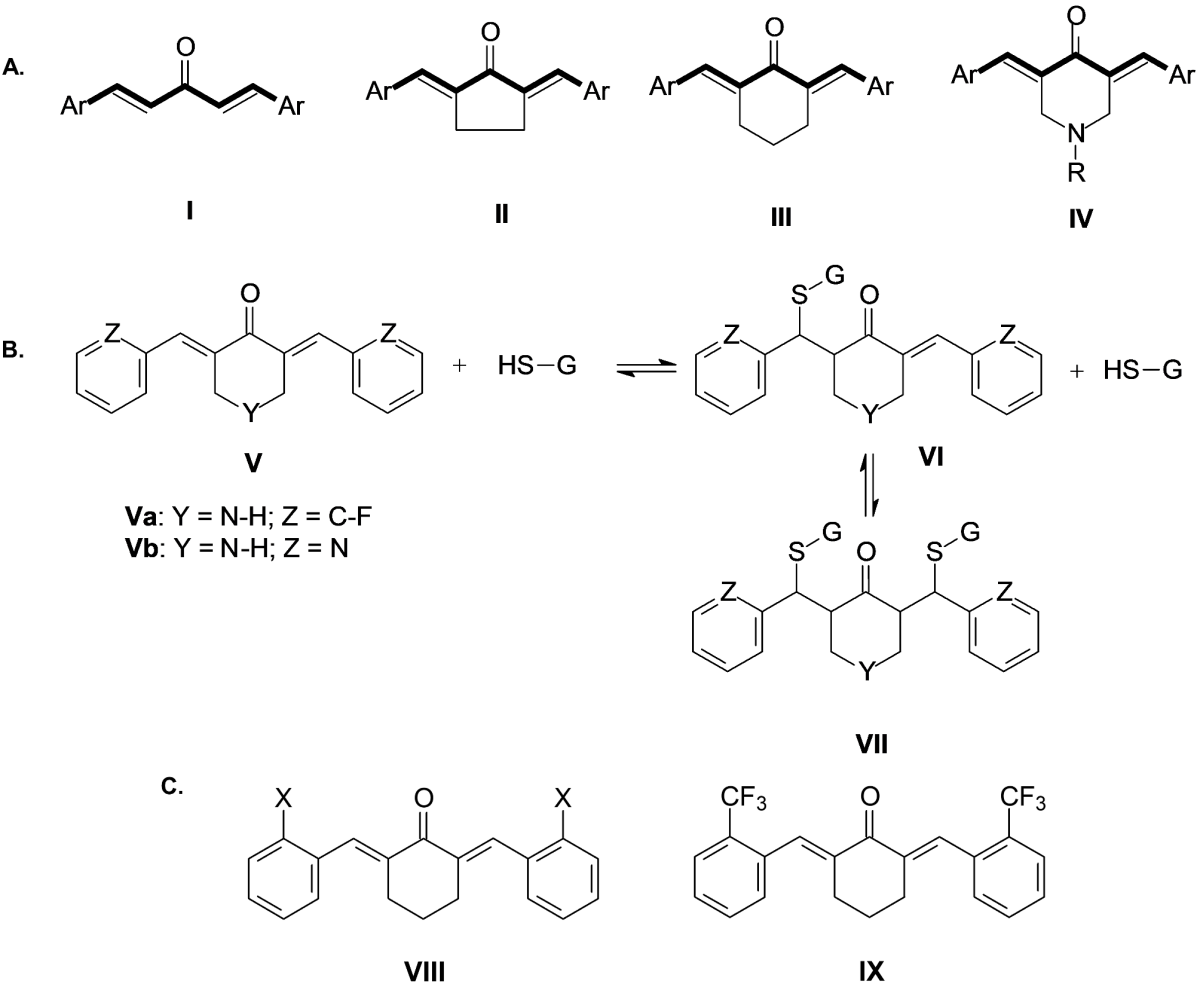
**Structures of monocarbonyl analogs of curcumin (MACs). A**. General structures of MACs (I-IV) containing a 1,5-diaryl-3-oxo-1,4-pentadienyl pharmacophore (I - “acetone”, II - cyclopentanone, III - cyclohexanone, IV − 4-piperidone), Ar = aryl, R = H, alkyl, aryl). **B**. In vitro reaction between MAC and glutathione (GSH) in acetonitrile/water, yielding a monoglutathione- and bisglutathione-adduct (Va: Y = N-H, Z = C-F, (3*E*,5*E*)-3,5-bis[(2-fluorophenyl)methylene]piperidin-4-one; Vb: Y = N-H, Z = N, (3*E*,5*E*)-3,5-bis(2-pyridylmethylene)piperidin-4-one). **C**. General structure of a symmetric MAC − (2*E*,6*E*)-2,6-bis(2-X-benzylidene)cyclohexanone (VIII), and the structure of (2*E*,6*E*)-2,6- bis[(trifluoromethyl)benzylidene)cyclohexanone (C66) (IX)

Inspired by the biological activity of curcumin, many research groups have tried to remove/alter its active sites. It has been proposed that important part of the biological activity of curcumin is stemming from the α,β-unsaturated ketone fragments, which act as a Michael acceptor. Additionally, it has been established that the main reason for the low chemical stability and bioavailability is the enolizable 1,3-diketone (β-diketone) moiety due to the reactivity of the enol form (Nelson et al. 2017). Many efforts have been undertaken to improve curcumin’s stability and bioavailability (structure-activity relationship (SAR) approaches) (Anand et al. 2008), (Dimmock et al. 1999; Liang et al. 2009; Arshad et al. 2017; Zheng et al. 2017). In order to keep the reactive fragment but to improve the bioavailability, new so-called monocarbonyl analogs of curcumin (MACs) have been prepared and investigated (Fig. 2) (Dimmock et al. 1993, 2003; Das et al. 2006, 2008; Zhao et al. 2013; Shetty et al. 2014; Liang et al. 2008; Kumar et al. 2017; Qian et al. 2015). These analogs contain the 1,5-diaryl-3-oxo-1,4-pentadienyl moiety with (in most cases) *E*,*E* stereochemistry of the double bonds. There is a wealth of information about their physicochemical properties and most importantly, they are synthetically accessible (via Claisen–Schmidt condensation) in high purity, and are stable, both in solid state and in solution (however, based on our experience, the solutions of these derivatives should be kept in the dark (or prepared in amber glassware), because they are susceptible to photochemical *E*-*Z* isomerization).

The key study by Liang et al. (2009) confirmed that the three categories of monocarbonyl analogs (I, II, and III, Fig. 2 A) have better hydrolytic stabilities compared to curcumin at pH 7.4 and 37 °C. In general, the cyclopentanone analogs (II, Fig. 2 A) are the most stable, followed by the cyclohexanone derivatives (III, Fig. 2 A). The hydrolytically least stable in the above-mentioned conditions are acetone-type analogs (I, Fig. 2 A). The conclusion of their SAR study was that the electron-withdrawing substituent at ortho (2) position of the benzene ring generally increased their bioactivity (cytotoxicity). Also, the higher electron-withdrawing capability of the substituents resulted in a stronger cytotoxic effect in tumor cells. From the tested symmetrical analogs with various EWG substituents (X = F, Cl, Br, or CF_3_) in ortho position in all three categories, the MACs with the CF_3_ group were the most potent. From the experimental experience gained in our labs, we have focused on cyclohexanone analogs (III, Fig. 2 A) because of their higher stability compared to group I MACs (I, Fig. 2 A) and their better solubility in various solvents compared to cyclopentanone type (II, Fig. 2 A) MACs. Moreover, the cyclohexanone system is conformationally more flexible compared to the cyclopentanone system, which could be beneficial in binding to relevant receptors.

Another important feature is that these cross-conjugated dienones are potent Michael-acceptors and have affinity for thiols (Das et al. 2009). The preferential reactivity of these MACs towards biologically relevant thiols, such as glutathione, has been pinpointed as the key factor for their versatile bioactivity. Moreover, it has been shown in vitro that certain MACs of curcumin (Va and Vb, Fig. 2B) form mono-glutathione adducts (VI, Fig. 2B) and bis-glutathione adducts (VII, Fig. 2B) and that this process is reversible (Sun et al. 2009). Another appealing feature of these derivatives is their tunability in terms of the electron density of the olefin moiety via incorporation of substituents on the benzene rings (Amslinger 2010; Al-Rifai et al. 2013; Amslinger et al. 2013).

One frequently employed approach to safeguard against in vivo metabolism involves insertion of electron-withdrawing functionality, such as the trifluoromethyl (CF_3_) group, into drug candidates (Filler et al. 1993). This group has a unique role in drug discovery chemistry, which may be witnessed by its presence in many approved medicines. Introduction of CF_3_ into molecules usually improves the binding by promoting electrostatic interactions with targets. Additionally, it increases the cellular membrane permeability, improves the metabolic stability of the drug, and may impart selective reactivities (Müller et al. 2007; Purser et al. 2008; Hagmann 2008). Recently, a synthetic method for introduction of CF_3_ group directly on the aryl ring has been reported, thus providing better flexibility in preparation (Nagib and MacMillan 2011). With the introduction of trifluoromethyl group which is EWG (electron-withdrawing group) with strong inductive effect at the (closest) ortho positions of the 2,6-dibenzylidenecyclohexanone, one effectively decreases the electron density of the β-carbon of the enone and makes it more electrophilic and more susceptible to attack from nucleophilic thiols. Also, the CF_3_ group is not sterically demanding and does not significantly distort the enone geometry of the 2,6 − 1,5-diaryl-3-oxo-1,4-pentadienyl (Fig. 2). The combining of two known pharmacophores (1,5-diaryl-3-oxo-1,4-pentadienyl and CF_3_) to a first approximation should be beneficial in terms of chemical and metabolic stability.

**Fig. 3:**
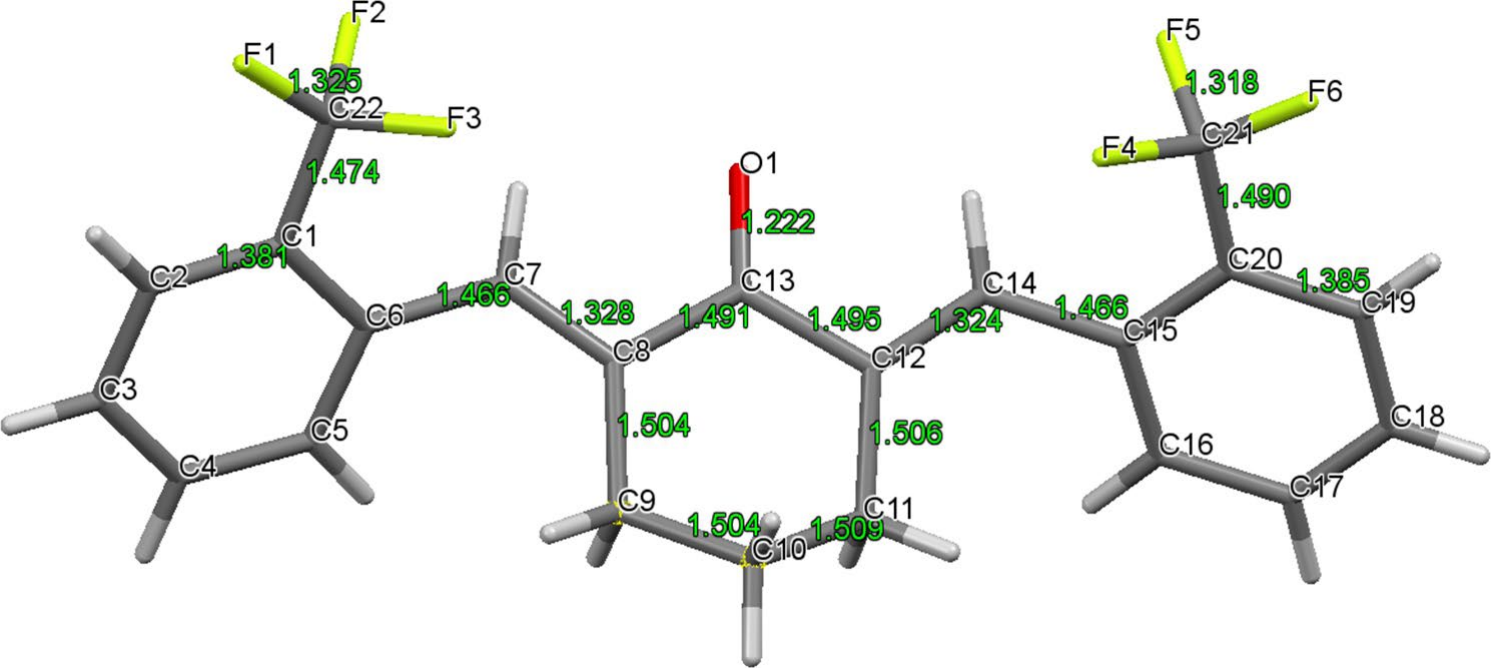
**Molecular structure of (2*E***,**6*E*)-2,6-bis[(2-trifluoromethyl)benzylidene]cyclohexanone (C66)**. The structure was obtained by recrystallization from methanol (slow evaporation at ambient temperature (41). The atom numbering of the molecular structure generated from the crystallographic file and the bond lengths are given in green color. The X-ray crystallographic data was obtained from the Cambridge Structural Database (ID, MUSHAL, deposition number 1,432,340)

Indeed, the experimental results showed improved chemical stability and pharmacokinetics of the resulting compound (2*E*,6*E*)-2,6-bis[(trifluoromethyl)benzylidene)cyclohexanone (C66) (Fig. 2 C), compared to curcumin (Liang et al. 2009). In this manner, a stable compound is obtained, with defined stereochemistry that can be reproducibly synthesized in high purity, and that is readily soluble in organic solvents. The *E*,*E* stereochemistry of the exocyclic double bonds, the conformation of the cyclohexanone ring, and the dihedral angles of the aryl rings have been confirmed by spectroscopic and crystallographic data (Fig. 3) (Zhang et al. 2015).

From the crystallographic data, it can be established that the C13-O1 bond length is 1.22 Å, which is in the range for the carbonyl group of aldehydes and ketones. Also, the C-C bond lengths in the cyclohexane ring are in the range of regular single bonds. The C7-C8 and C12-C14 bonds have bond lengths of 1.328 Å and 1.324 Å, which fall in the range of typical double bonds; they have E stereochemistry. On the other hand, both C6-C7 and C14-C15 bonds have the same length (1.466 Å), which falls between single and double bonds. The shortening of these bonds is typical for an enone structure, which is caused by the neighboring conjugated double bonds. The enone system is not perfectly planar, and the phenyl groups are also twisted with respect to the enone (13° and 37°). In this arrangement, the electrophilic β-carbons (C7 and C14) of the cross-conjugated enone are accessible to potential nucleophiles. It should be noted that C66 has conformational flexibility in solution, which can be beneficial regarding its binding to active sites of enzymes.

**Fig. 4:**
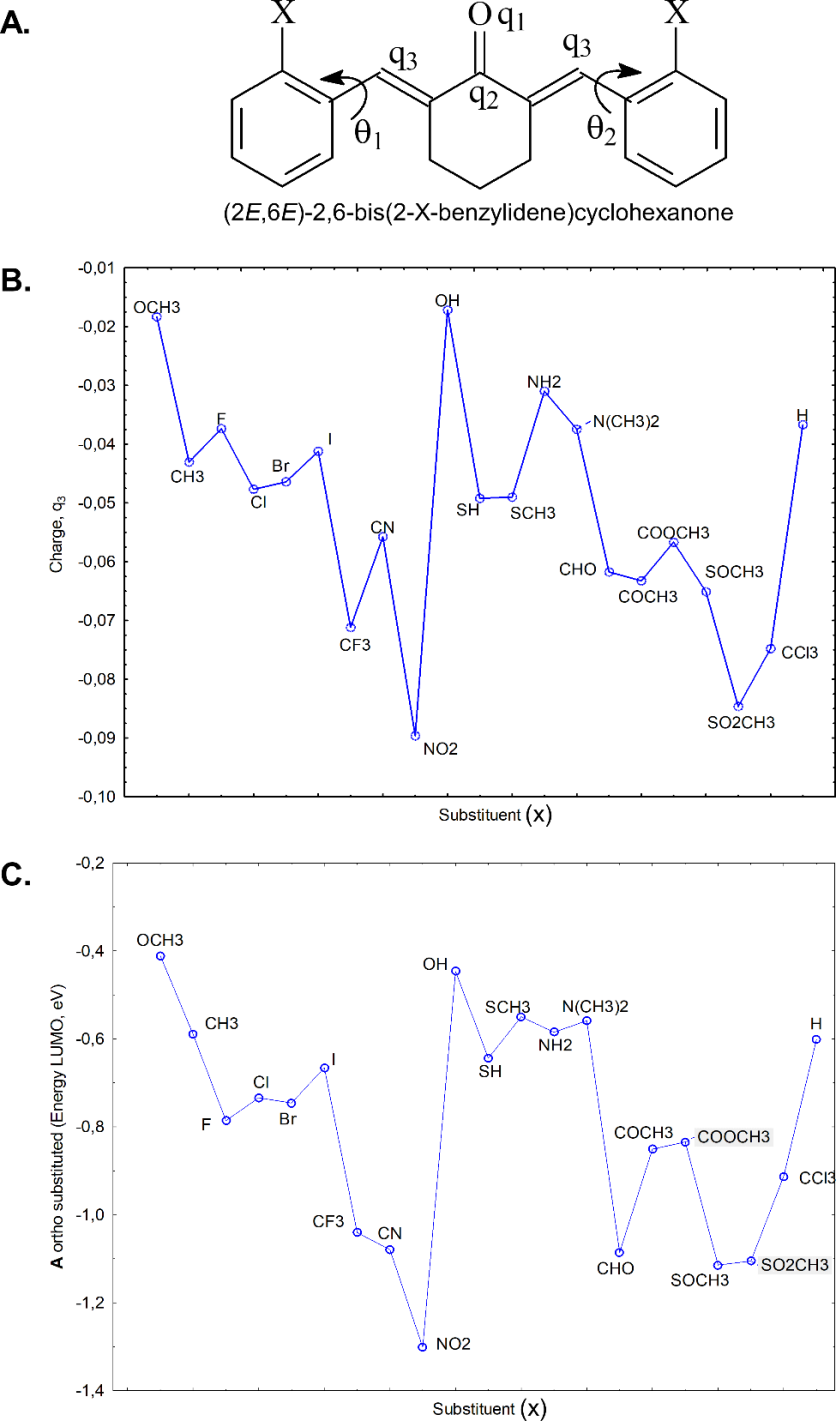
**Quantum mechanical parameters (semi-empirical PM3 method) of different symmetrical MACs (bis (ortho-substituted 2,6-dibezylidenecyclohexanones), including C66 (X = CF**_**3**_**). A.** General structure of a symmetrical MAC [(2*E*,6*E*)-2,6-bis(2-X-benzylidene)cyclohexanone], where X is the substituent atom/group (e.g., Br, CF_3_, NO_2_); q_1,2,3_ represents charge density on the respective atom; and θ_1,2_ is the torsional angle between the respective bonds. **B**. Graph showing the charge density of the beta-carbon of the enone, q_3_, depending on the substituent atom/group. The presence of a strong electron-withdrawing substituent leads to a decrease in the charge density, which in principle makes the MAC a better Michael acceptor. **C**. Graph showing the energy of the lowest unoccupied molecular orbital, E_LUMO_, as a function of the substituent atom/group. E_LUMO_ is related to the electron-accepting capability of the MAC

In order to pinpoint the pertinent structural features that dictate the reactivity towards thiols as previously reported (Hadzi-Petrushev et al. 2018), we have employed simple computational chemistry (semi-empirical PM3 method) on these MACs with appropriate ortho substituents. The emphasis was placed on electronic properties, conformation, torsional angle of the aryl group, and especially on the energy of the frontier molecular orbitals, the highest occupied molecular orbital (E_HOMO_), and lowest unoccupied molecular orbital (E_LUMO_) (Das et al. 2008). These quantum chemical descriptors can be taken as a useful initial guide in the search for new compounds that may exhibit antinflammatory and/or antioxidant activity (Sklenar and Jäger 1979; Karelson et al. 1996; Zoete et al. 2004; Shakman and Mazziotti 2007). From the quantum mechanical calculations presented graphically (Fig. 4B), it can be seen that the presence of strong EWG such as NO_2_, SO_2_CH_3_, CCl_3_, CN, CF_3_, leads to a decrease in the charge density (on q_3_), which in principle makes these derivatives better Michael acceptors in reactions with thiols. On the other hand, strong electron donating groups (EDG) such as NH_2_, N(CH_3_)_2_, OCH_3_, OH, have the opposite effect on the charge density of the beta-carbon (q_3_) of the enone, and consequently should be less reactive (less electrophilic) towards thiols. Similar trends can be seen in terms of E_LUMO_, which indicates the electron accepting capability of a compound (Fig. 4 C). C66 has comparable properties in terms of charge density on q_3_ and E_LUMO_ with dicyano analogs (X = CN), but is expected to be more chemically stable. It can be seen that based on the charge on the position q3 (of the Michael acceptor) and the E_LUMO_, C66 is expected to be more reactive towards (biologically relevant and other model) thiols compared to the parent compound 2,6-dibezylidenecyclohexanone (R = H) and to compounds with EDG.

The advantage of introducing the CF3 group is that this group is EWG by inductive effect, it is not sterically demanding, and affects the energy of LUMO to a lesser extent than the other electron-withdrawing groups, which exert their influence through resonance. The energy of LUMO for C66 (X = CF3) is less negative than the other strong EWG (such as NO2, CN, CHO, SO2CH3, etc.). Compounds that are strong electron acceptors, such as 2,6-bis(2-nitrobenzylidene)cyclohexanone, can alter certain biologically relevant redox processes, which could cause unwanted side effects (Nepali et al. 2019).

Analogously, E_HOMO_ indicates electron donating power of a compound, and compounds with EDG, such as NH_2_, N(CH_3_)_2_, OCH_3_, OH, are expected to have antioxidant properties. Usually, they suppress the formation or stop the propagation of free radicals and other reactive species. There is a study where the EHOMO of curcuminoids (1 and 3, Fig. 1) and MACs of type I, II, and III (Fig. 2 A) with electron-donating (OH and OCH3) groups are correlated and ranked based on their enzyme-inducing capability (Zoete et al. 2004). The presence of OH and/or OCH3 has a strong influence on the EHOMO and electron-donating properties. The 2,6-diarylidenecyclohexanones with EWG, such as C66, have pronounced anti-inflammatory activity, while the analogs with EDG generally have superior antioxidant effects. However, the electron density on the benzene ring is higher in these analogs with EDG, and they are more prone to enzymatic metabolism in vivo by cytochrome P450 oxidases (Montellano 2005). Hence, compared to curcumin and to some other curcuminoids, there are several advantages of C66: synthetic accessibility, structural simplicity, improved chemical stability (in vitro and in vivo), increased electrophilicity of the enone, presence of two reactive electrophilic centers, and moderate electron accepting capacity. In our lab, we have been preparing C66 and applying it in several studies (Hadzi-Petrushev et al. 2018), (Stamenkovska et al. 2020), (Hadzi-Petrushev et al. 2019), assessing its antioxidative and anti-inflammatory activity. All the above-mentioned studies lead to the notion that C66 is an ideal compound for testing as a treatment for different pathophysiological conditions. In the following sections, we review the literature concerning the application of C66 in resolving diabetes-associated cardiovascular and renal complications in animal models.

## C66 and diabetes-associated complications

### Effects of C66 on the prevention of diabetes-associated vascular damages

Hyperglycemia is the key link between diabetes and high oxidative stress that is implicated in the pathophysiology of vascular abnormalities caused by diabetes (Maritim et al. 2003), (Fiorentino et al. 2013). Excessive generation of reactive oxygen species (ROS) has been identified as an early pathogenic component in diabetic aortic damage, which can be mitigated by enhanced endogenous antioxidant capacity (Li et al. 2018a, b, c).

Chronic inflammation, as one of the main factors in the progression of diabetes and its complications, causes tissue damage and leads to the generation of new vascular structures via oxidative stress, apoptosis, endothelial dysfunction, and fibrosis (Wang et al. 2014a, b, c), (Liu et al. 2014; Li et al. 2018a, b, c) reported elevated levels of proinflammatory (TNF-1α, MCP-1), aortic fibrosis (CTGF, TGF-β1), apoptosis (caspase-3), and oxidative stress (3-nitrotyrosine (3-NT), 4-hydroxynoneal (4-HNE)) markers in the aorta of diabetic mice. Treatment with C66 or deletion of JNK2 (JNK2^−/−^) in diabetic mice resulted in a reduction of the increased expression of inflammatory and fibrosis markers (Li et al. 2018a, b, c). In fact, treatment with C66 had no additional effect on JNK2^−/−^ diabetic mice, suggesting that C66 protection is based on the suppression of JNK2 (Parsamanesh et al. 2018), (Li et al. 2018a, b, c). As a member of the mitogen-activated protein kinase family, JNK2 influences multiple cellular stress responses, including inflammatory responses, oxidative stress, cell death, cell survival, and protein expression, in various tissues of diabetic animals (Liu et al. 2014; Zhou et al. 2016; Jiao et al. 2015; Fan et al. 2012). JNK was found to be inhibited by curcumin, which is a potent protector against cardiovascular disease (Fiorillo et al. 2008; Stamenkovska et al. 2021; Pan et al. 2013) found that C66 has a high affinity for JNK2 binding, which potentiates its anti-inflammatory effects. In addition, Li et al. (2018a, b, c) have found that JNK2 deletion is associated with reduced aortic inflammation, oxidative stress, apoptosis, and fibrosis, caused by diabetes, but significant changes in the aorta of diabetic JNK2^−/−^ mice were not reported after C66 treatment. These data imply that protection against aortic damage caused by diabetes is mediated by C66-induced JNK2 inhibition. Hence, reducing JNK2 activity by C66 can be suggested as an effective supplementary technique in the treatment of diabetes.

**Fig. 5:**
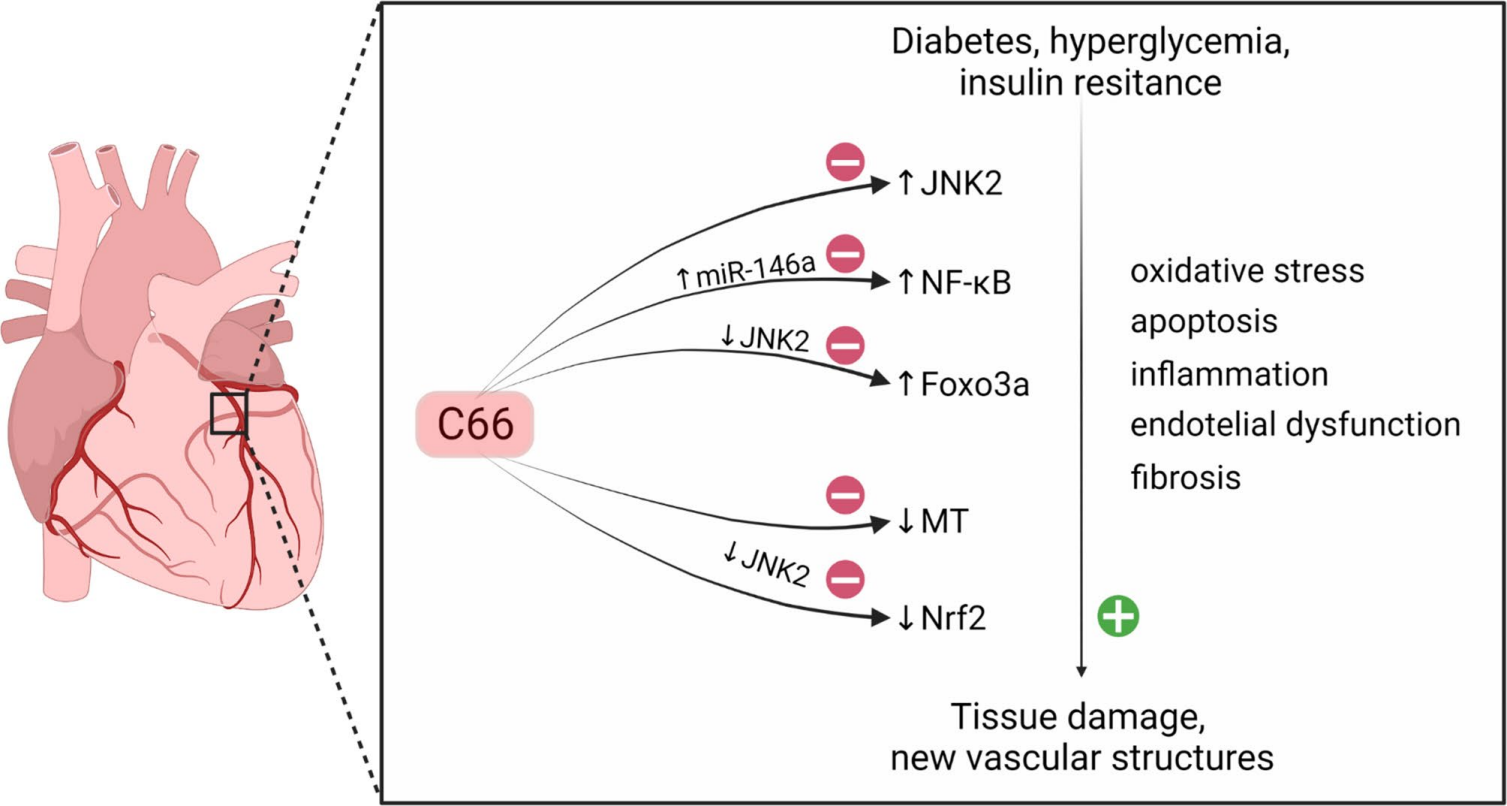
**Effects of C66 in the reduction of diabetes-associated damage to the cardiovascular system.** The full names of the figure components are given in the list of abbreviations

Furthermore, Huang et al. (2020) showed that when human umbilical vein endothelial cells (HUVECs) were incubated in high glucose (HG) (25 mM) medium, C66 caused dose-dependent (0–5 µM) suppression of the phosphorylated p-65 (p-p65)-induced expression. Interleukin 1 receptor-associated kinase (IRAK1), as a key adapter downstream kinase of the toll-like receptor (TLR) superfamily, mediates HG-induced NF-kB activation by phosphorylation and degradation of the IkB protein in the proteasomes (Hayden and Ghosh 2004; Huang et al. 2020) also reported that treatment with HG (25 mM) caused a significant reduction in microRNA miR-146a expression, which was restored by the administration of C66. Additionally, the authors suggested that pre-treatment with hsa-miR-146a antagonist, completely abolishes C66-induced miR-146a upregulation in HUVECs incubated with HG (Huang et al. 2020). C66 at doses up to 5 µM caused a significant limitation of the HG-amplifying effect of IRAK1 and p-p65 expression in HUVECs, but these effects were reversed with anti-miR-146a treatment (Huang et al. 2020). Therefore, it is apparent that C66 can counteract HG-induced NF-κB activation in HUVECs by stimulating miR-146a expression (Fig. 5).

### Effects of C66 on the prevention of diabetes-associated cardiac damage

As mentioned above, excessive production of ROS results in oxidative stress, which can contribute to the development and the progression of a wide range of diabetic complications, including heart diseases (Weseler and Bast 2010). It is well known that diabetes-induced disulfide bond formation in the myocardial endoplasmic reticulum (ER) causes oxidative stress and apoptotic cell death (Nakamura et al. 2001). Increased production of ROS and nitric oxide (NO) has also been linked to impaired cardiac endothelial cell function (Senoner and Dichtl 2019). Thereby, active myocardial ER stress is followed by depletion of ER-Ca^2+^ stores caused by overproduction of NO by inducible NO synthase (iNOS) (Wang et al. 2014a, b, c). In this regard, Cai et al. (2005) found that diabetic mice have higher levels of oxidative damage and oxidative stress in their myocardial ER. Actually, this group has shown that at the level of the myocardium, in the same manner as in the aorta, C66 and JNK inhibition causes reduction of the indicators of oxidative stress, ER stress, and in the levels of proteins of the caspase family, such as caspase-12 and caspase-3. Furthermore, Cai et *al.* (Cai et al. 2005) have confirmed that C66-induced reduction in the JNK phosphorylation could be mediated by overexpression of metallothioneins (MT) resulting in protection of the heart from diabetic impairment. Several studies (Cai et al. 2006; Miao et al. 2013; Song et al. 2005) demonstrated that MT provide antioxidant protection of the myocardium from different disorders, including diabetes (Velic et al. 2013), (Ye et al. 2003). Namely, in parallel to the pathological heart damage, diabetes causes decreased expression of MT in the heart (Cai et al. 2005). Furthermore, Miao et al. (2013) reported reduced early cardiac cell death by regulation of the mitochondrial oxidative stress, followed by suppressed development of diabetic cardiomyopathy in transgenic diabetic mice with overexpressed cardiac MT. In addition, Xu et al. (2009) reported MT-induced decrease in diabetes-related cardiac ER stress, accompanied by reduced cell death, most likely due to their antioxidant effect. Two studies reported existence of a potential link between JNK phosphorylation and MT expression, implying involvement of JNK in the diabetes-induced apoptotic cell death (Lau et al. 2006), (Qu et al. 2006). Another study revealed that MT cause prevention of JNK-provoked cell death by neutralizing ROS (Peng et al. 2007). These data imply that the diminished impact of diabetes on the apoptotic processes as a result of the treatment with C66 is associated with overexpression of MT, which could be related to the overall control of the JNK signaling, however, whether MT directly inhibits JNK phosphorylation, is not clear yet. Although further studies are needed to understand the underlying processes, it is evident that the inhibition of JNK, which mediates the protective effects of C66 against diabetes-induced cardiac damage, is based on the suppressed ER stress and increased MT expression in cardiac myocytes. However, the specific mechanism by which C66 and JNK inhibition retain the MT expression is unclear, thus further studies are needed in this area.

Recent C66-diabetes-related studies give indications of another important diabetes-associated factor known as the nuclear factor erythroid 2-related factor 2 (Nrf2) (Kensler et al. 2007). It was shown that Nrf2 possesses therapeutic benefits in the settings of diabetic complications via contributing to the induction of antioxidant enzymes synthesis. When Nrf2 is silenced, endothelial progenitor cell motility, proliferation, and secretion, is reduced, while oxidative stress and cell aging are increased (Wang et al. 2018). In opposite, it was found by Li et al. (2018a, b, c) that overexpression of Nrf2 causes reduction in the production of ROS and in the expression of inflammatory cytokines in HG cultured endothelial progenitor cells (Li et al. 2018a, b, c) also reported that diabetic JNK2^−/−^ mice treated with C66 show significant changes in Nrf2 expression compared to diabetic JNK2^−/−^ mice. Thus, JNK2 was established as a key regulator of the Nrf2 function, indicating at the same time its involvement in the C66-induced protection. Nrf2 participates in the regulation of cell detoxification and redox status regulation via promotion of the expression of many antioxidant genes. Hence, the processes of JNK2 activation and Nrf2 inhibition are associated with oxidative stress induced by diabetes, while the process of JNK2 suppression is related to the increased Nrf2 expression (Li et al. 2018a, b, c). Basically, it seems that treatment with C66 or deletion of JNK2 stimulate expression of Nrf2. Li et al. (2018a, b, c) also examined the level of expression of the Nrf2 downstream genes, including *HO-1*, *NQO1*, and *SOD1*, and found that these antioxidant enzymes are significantly upregulated in the C66-treated diabetic mice. Hence, the preliminary conclusion should be that the antioxidant effect of C66 is Nrf2-mediated, but mainly dependent on JNK2.

In the search for additional C66-diabetes-associated molecular players, forkhead box O3a (FOXO3a) transcription factor was reported to be an essential downstream signaling effector of apoptosis (Lam et al. 2006). In this direction, the report of Sunters et al. (2006) suggested a JNK-associated FOXO3a-dependent apoptosis, followed by enhancement in the nuclear translocation of FOXO3a, and pro-apoptotic gene expression. The same group published that activation of FOXO3a is controlled by phosphoinositide 3-kinase (PI3K)/AKT serine/threonine kinase 1 (Akt) signaling pathway. Actually, the subfamily of FOXO, as a downstream target of the PI3K/Akt signaling pathway, were found to be involved in the regulation of cell cycle homeostasis, apoptosis, differentiation, metabolism, migration, oxidative stress, DNA damage, and other important cellular processes (Lam et al. 2006). In a similar manner, the HG, as one of the hallmarks of diabetes, causes reduction of the nuclear translocation of phosphorylated Akt (p-Akt), which is the reason for further promotion of FOXO3a-dependent apoptosis during diabetic cardiomyopathy (Sunters et al. 2006). Besides its ability to reduce p-Akt and increase FOXO3a activity, JNK is simultaneously responsible for the dynamics of the nuclear localization and FOXO3a control (Burillo et al. 2021). It is therefore logical to imply that FOXO3a activation competes with the JNK (probably via JNK2) and the PI3K/Akt signaling pathways, both of which have been shown to be reduced in well-controlled diabetes (Li et al. 2018a, b, c). Hence, cross-linking between these signaling pathways as well as FOXO3a inactivation could be of critical importance in cardiac apoptosis. It should be noted that diabetes causes reduction in the level of phosphorylation of PI3K, Akt, and FOXO3a, which is altered by C66 in wild-type diabetic, but not in JNK2^−/−^ diabetic mice, suggesting that C66-induced activation of these enzymes mediates suppression of the JNK2 activity.

Finally, based on the data from Li et al. (2018a, b, c) the cardioprotective effects of C66 appear to be dependent on JNK2 (Fig. 5). That is, C66 causes inhibition of JNK2, which reduces cardiac inflammation, fibrosis, oxidative stress, and apoptosis, in the settings of diabetic cardiomyopathy. Precisely, the mechanism involved in the protective effect of C66 alongside inhibition of JNK2, involves overexpression of Nrf2. Additionally, Li et al. (2018a, b, c) established a link between C66, JNK, and PI3K/Akt signaling in FOXO3a-dependent regulation of diabetes. These findings are expected to assist in the development of preventive and therapeutic methodologies for the suppression of diabetic cardiomyopathy.

### Effects of C66 on the prevention of diabetes-associated kidney macrophage infiltration

The chronic inflammatory response in diabetics has been shown to induce infiltration and accumulation of macrophages in the kidney (Tesch et al. 2010) According to Yonemoto et al. (2006) besides macrophage infiltration, diabetic nephropathy (DN) is characterized by mesangial matrix dilatation and interstitial fibrosis. A specific pathological manifestation in patients suffering from DN was the induced macrophage production of inflammatory cytokines such as IL-1β, IL-6, IL-12, IL-18, TNF-α, IFN-γ, and MCP-1, (Hirata et al. 1998), (Wen et al. 2006) via NF-κB-dependent pathway that subsequently lead to albuminuria and renal fibrosis.

**Fig. 6:**
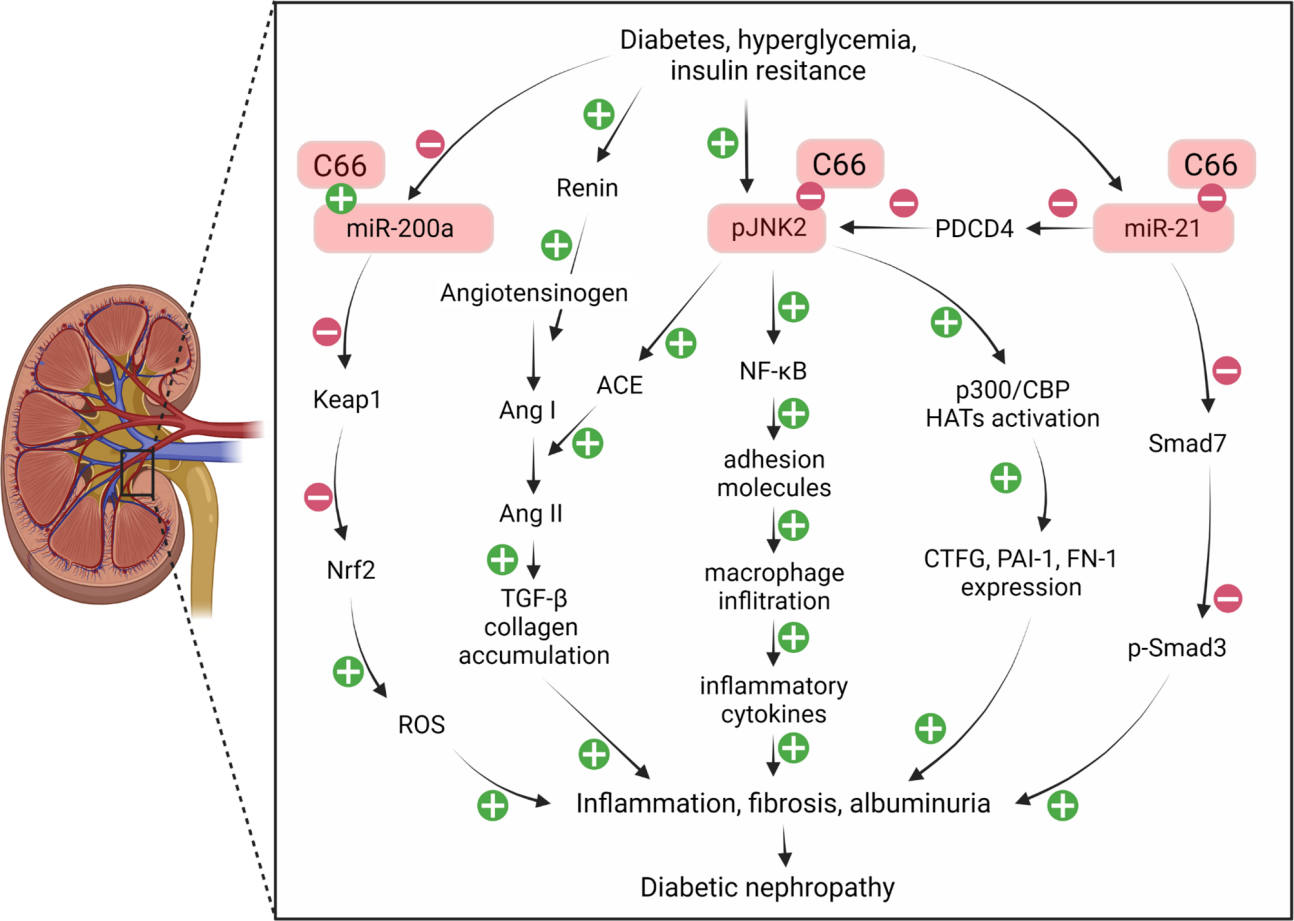
**Effects of C66 in the reduction of diabetes-associated kidney damage.** The full names of the figure components are given in the list of abbreviations

Recently, it was also shown that metabolic products from diabetic impairment of the kidney may promote macrophage recruitment by inducing expression of MCP-1 and other cell adhesion molecules (Yang et al. 2007). Thus, infiltrated macrophages in the inflamed kidney initiate production of profibrotic cytokines such as TGF-β, which play a key role in progressive renal fibrosis (Yang et al. 2007) In their studies, Park et al. (2000) and Kosugi et al. (2009) confirmed that ICAM-1, VCAM-1, and MCP-1 play a very important role in the pathogenesis of diabetic nephropathy by inducing inflammatory macrophage infiltration (Park et al. 2000), (Kosugi et al. 2009). Furthermore, Pan et al. (2013) reported diabetes-induced increased renal expression of VCAM-1, ICAM-1, and MCP-1, associated with macrophage infiltration, adhesion, and renal fibrosis. But, most importantly, these two groups have shown that both in vitro and in vivo, the aforementioned increases in various factors in the settings of HG are attenuated as a result of the treatment with C66. Hence, the anti-inflammatory effect of C66 in diabetes-associated renal impairment appears to be the result partially of the suppressed expression of VCAM-1, ICAM-1, and MCP-1 in the renal epithelium. The studies of Pan et al. (2013) exploring the transcriptional mechanism by which C66 causes inhibition of the diabetes-induced adhesive molecules expression have shown renal IkB degradation and p65 nuclear translocation in cultured renal epithelial cells isolated from diabetic mice. Having in mind that NF-kB initiates renal inflammatory processes by regulating the gene expression of cytokines, chemokines, and adhesive molecules, it is logical to predict that VCAM-1 and ICAM-1 expression would be NF-kB dependent (Navarro-González et al. 2011). Two independent groups have also found that NF-κB blockade is accompanied by inhibition of the MCP-1 gene expression in epithelial cells from the diabetic kidney (Lee et al. 2004), (Wang et al. 2000). In relation to the protective role of C66, Pan et al. (2013) reported C66-induced inhibition of NF-κB in renal epithelial cells, which is followed by a highly significant reduction in the HG-induced expression of ICAM-1, VCAM-1, and MCP-1. Based on all above, it can be expected that the C66-induced reduction of the expression of the listed adhesive molecules in diabetic kidneys is accompanied by inactivation of the NF-κB. One of the interesting findings of the study of Pan et al. (2013) is that all three MAPK subfamilies (ERK, p38, and JNK) are implicated in the HG-induced NF-kB activation (to a different extent). Although there are several reports of cross-linking between MAPKs and NF-κB, it appears that MAPKs modulate the expression of inflammatory cytokines in the HG-induced renal cells. The same group further confirms that JNK is a critical upstream molecule in NF-κB-induced signaling and plays a very important role in the HG-induced renal inflammation. This is the only report to date of HG-induced JNK-mediated expression of VCAM-1 and ICAM-1, indicating the importance of JNK in the production of renal cytokines (Pan et al. 2013). Consistently, it has been also reported that the treatment with C66 in diabetic mice may inhibit NF-kB activation and cause reduction in the ICAM-1, VCAM-1, and MCP-1 production in renal tissue, through JNK-mediated mechanisms (Fig. 6) (Pan et al. 2013). All of the studies mentioned above potentiate another beneficial effect of C66, associated with a significant reduction of the macrophage infiltration in the diabetic kidney, followed by a decrease in circulating creatinine and attenuation of pathological indices of renal injury.

### Effects of C66 on the prevention of diabetes-associated renal fibrosis and nephropathy

Numerous studies in the last decade have shed light on the mechanisms by which curcumin acts on renal fibrosis, as well as the mechanisms by which it alleviates the problems of DN (Sun et al. 2017). Such effects are accompanied by reduction of inflammatory factors (MCP-1, NF-κB, TNF-α, IL-1β, COX-2, and CAV-1), stimulation of the expression of anti-inflammatory factors (HO-1, M6PRBP1, and NEDD4), as well as by targeting TGF-β/SMADs, MAPK/ERK, and PPAR-mediated pathways in animal models. Despite the fact that curcumin may not be able to completely repair kidney damage caused by diabetes-associated nephropathy, the idea that curcumin may affect renal fibrosis was partially confirmed by various animal studies (Sun et al. 2017). Therefore, many authors point to the need for prospective studies that will explain the mechanisms by which curcumin acts to alleviate renal fibrosis. However, it is well known that its low bioavailability limits its use for this purpose. Fortunately, at least one of its derivatives with acceptable bioavailability (such as C66) may offer new options in the treatment of renal fibrosis, which with its new chemical structure will define the basis for the synthesis of new pharmaceutical formulations against renal fibrosis.

Recent studies have also shown that the p300/CBP complex, together with the diabetes-induced modifications of histone acetylation, is likely to participate in the development of diabetes-associated complications. In this regard, the study Wang et al. (2015) of streptozotocin (STZ)-induced diabetic mice found that diabetes was associated with a significant increase in total histone acetyltransferases (HATs) activity. In fact, the authors corroborated that the treatment of diabetic mice with JNK inhibitors (JNKi), yielded similar results to those obtained with C66 treatment. JNK activation is associated with p300 recruitment followed by acetylation of the H3 and H4 histones (Tsai et al. 2012). Accordingly, in diabetic mice, C66 has been shown to reduce the diabetes-associated increase in p300/CBP expression and HATs activation by JNK inactivation, whereby the subsequent histone hyper-acetylation causes specific increase in p300/CBP-mediated accumulation of CTGF, PAI-1, and FN-1 gene promotors (Fig. 6). Thus, Tsai et al. (2012) reported for the first time that C66 may act as a potent preventive agent against diabetes-associated renal fibrosis and renal impairment in STZ-induced mice models of diabetes. Specifically, they showed that a 3-month course of C66 treatment caused prevention of the diabetes-associated renal JNK signaling, accompanied by subsequent upregulation of the renal fibrotic signaling. The consistency of this protective effect is primarily due to the ability of C66 to suppress p300/CBP activation through long-term epigenetic modifications, which may affect the “metabolic memory” that may underlie diabetes itself (Karukurichi et al. 2010), (Villeneuve et al. 2011). Another important benefit of studying C66 interactions with JNK and HATs pathways is that these pathways were highlighted as new potential targets of therapeutic strategy in combating the development of diabetic complications, particularly in the prevention of DN. Аlthough Wang et al. (2015) clearly indicate the dominant role of the JNK pathway in the development of DN and its prevention by C66 and JNK inhibitors, the very limited characteristics of C57BL/6J mice as the DN model used in these studies probably mask other signaling pathways affected by the use of C66 and/or JNK inhibitors (Noonan and Banks 2000). Therefore, the use of a mouse strain with a higher DN sensitivity, such as 129 SvEv mice, Hartner et al. (2003) may further assist in obtaining more information regarding C66- and/or JNK-mediated prevention of the early and late features of DN.

Two factors (oxidative stress and high blood pressure) have a special place in the study of diabetes-associated renal nephropathy, since the deterioration of each of them significantly worsens and accelerates the pathogenesis of diabetic nephropathy (Wu et al. 2016; Keshari et al. 2015; Li et al. 2014). In their study, Uruno et al. (2013) showed that the Keap1-Nrf2 system controls antioxidant redox signaling, implying its key role in the interaction with curcumin. Furthermore, Esatbeyoglu et al. (2012) confirms that curcumin has ability to disrupt the binding between Keap1 and Nrf2, leading to release of Nrf2. However, the fact that curcumin suppresses only Keap1 expression, (Soetikno et al. 2013a, b) suggests that it has ability for partial modification of the Keap1-Nrf2 complex. In this direction, Wu et al. (2016) have shown that C66 reduces the expression of Keap1 mRNA and its product Keap1 protein. In addition to modification of the Keap1-Nrf2 complex, reduced expression of Keap1 by curcumin may also cause Nrf2 induction (Wu et al. 2016). Furthermore, miR-200a was identified as a mediator between C66 and Keap1 mRNA by Eades et al. (2011) who at the same time found that miR-200a induces Keap1 mRNA degradation. Moreover, Wu et al. (2016) have shown that the C66-induced miR-200a stimulation as a result of the reduced Keap1 expression is indirectly accompanied by NRF2 activation. Finally, Zheng et al. (2011) showed that the Nrf2-specific agonists [(sulforaphane (SFN) and cinnamic aldehyde (CA)] do not have protective effects in Nrf2-null diabetic mice suffering from DN. Nevertheless, it is worth to note that even in absence of Nrf2, C66 still provides partial protection from DN. The difference between C66 and the other two aforementioned Nrf2 activators is probably due to the different lines of action. Indeed, it seems that SFN and CA, both provide protection exclusively via Nrf2, which was corroborated with the absence of DN preventive effect in the Nrf2-null diabetic mice (Soetikno et al. 2013a, b). Unlike SFN and CA, Yonggang et al. (Wang et al. 2014a, b, c) have shown that C66 has an additional function (Soetikno et al. 2013a, b), which is different from the upregulation of Nrf2 (Kang et al. 2007), (Balogun et al. 2003). On the other hand, the ability of curcumin to interact with miR-21, was found to play very important role in the pathogenesis of DN (Dey et al. 2011; Wang et al. 2014a, b, c; Zhong et al. 2013), probably, due to the miR-21’s involvement in the regulation of TGF-β-SMAD and PDCD4-JNK signaling pathways. Essentially, miR-21 causes reduction in the *Smad7* and *Pdcd4* mRNA expression by degradation of *Smad7* or *Pdcd4* mRNA, (Wu et al. 2016) leading to inhibition of the SMAD3 and JNK phosphorylation, (Bitomsky et al. 2004), (Chen et al. 2011) which is further associated with the increased expression of the renal fibrotic and inflammatory genes (Chen et al. 2011; Chung et al. 2010; Ai et al. 2015; Ishida et al. 1999; Bennett et al. 2001). Тhis is in the context of Wu et al. (2016) results according to which Nrf2 inhibition completely eliminates the protective role of C66 from oxidative damage, while C66 still protects against renal fibrosis. Additionally, C66-induced miR-21 inhibition negatively alters the TGF-β-SMAD3 and PDCD4-JNK signaling pathways, causing albuminuria even in the absence of Nrf2, confirming the inhibitory role of C66 on miR-21, completely different from that of Nrf2 induction. DN-associated miRNAs inhibition, attracted great interest in the last few years. The advantage of C66 is due to its involvement in the functional regulation of Nrf2, alongside its role in the regulation of the miR-21 reduction (Fig. 6). The process of Nrf2 activation already was used in different clinical trials. Numerous studies from the last decade (Hall and Bhalla 2014; Gold et al. 2012; Ellison 2014) suggest that the dosage of the Nrf2 agonists should be carefully selected. The fact that the effectiveness of C66 doses used for this purpose is much better than those of natural curcumin (20 to 80 times), makes it a unique tool in future clinical trials.

In their studies with STZ-induced diabetic rat models, Kato et al. (1999) and Kelly et al. (2002) showed that components of the renin-angiotensin system, including angiotensinogen, renin, angiotensin-converting enzyme (ACE), and angiotensin II (Ang II), are abnormally increased. Pan et al. (2014) confirmed that administration of C66 fosters negative regulation on the HG-induced expression of ACE, followed by subsequent Ang II increase. Additionally, two different studies (Day et al. 2001), (Eyries et al. 2002), also suggested that such an increase in ACE mRNA transcription is mediated by MAPKs and protein kinase C. HG was shown to significantly stimulate phosphorylation of MAPK, mediating macrophage renal infiltration followed by renal dysfunction (Zhou et al. 2012). In vitro studies examining the activation time of the MAPK during HG stimulation showed that MAPK phosphorylation is activated in a very short time (15 min), which classifies it as the upstream signal molecule of the HG-induced ACE-TGF-β 1 signal cascade. The employment of MAPK-specific inhibitors helps in the elucidation of the HG-induced overexpression of ACE and TGF-β1 in renal tubular cells, corroborating that MAPKs participate in the HG-stimulated ACE overexpression. Furthermore, the MAPKs appear to mediate the HG-induced RAS activation. On the other hand, according to Pan et al. (2014) the specific MAPK inhibitors do not show an inhibitory effect on renin mRNA expression. Adequate in vivo data confirms that administration of C66 causes inhibition of the ACE expression and subsequent serum Ang II levels, while upstream renin and angiotensinogen levels remain unaffected in diabetic kidneys. The fact that C66 causes significant inhibition of the HG-induced MAPK phosphorylation (in vitro and in vivo), represents another confirmation that MAPK are molecular targets of C66 (Pan et al. 2014). This new mechanism also implies reno-protective effects of C66 in diabetes-associated nephropathy. Consistently with previously published data about diabetic rats (Pan et al. 2012), the treatment with C66 in the settings of DN has been found to cause significant reduction in the renal accumulation of collagen and in the circulating pathological indices of renal impairment. The established pharmacological effects of C66 are associated with its MAPK-dependent inhibition of the renal ACE, Ang II, and TGF-β1 increase. More importantly, the new concept that the MAPK signal pathway is involved in the HG-induced RAS activation and ACE overexpression due to the development of DN suggests that one of the strategies of DN treatment should be reduction in ACE expression by inhibition of MAPK (Fig. 6). Surely, everything mentioned above, lays down the basis for designing new preclinical and even clinical studies, which will target the C66 interactions with MAPK-dependent signaling mechanisms. The complete elucidation of these mechanisms will help to provide a clearer perspective of the protective effects of C66 in the prevention of diabetes-associated renal fibrosis and nephropathy.

## Conclusion

The cardiovascular protective effect of C66 against diabetes-induced oxidative damage is Nrf2-mediated, but mainly dependent on JNK2. In general, C66 causes inhibition of JNK2, which reduces cardiac inflammation, fibrosis, oxidative stress, and apoptosis in the settings of diabetic cardiomyopathy. C66 exerts a powerful antifibrotic effect through reducing inflammation-related factors (MCP-1, NF-κB, TNF-α, IL-1β, COX-2, and CAV-1) and inducing the expression of anti-inflammatory factors (HO-1 and NEDD4), as well as targeting TGF-β/SMADs, MAPK/ERK, and PPAR-γ signaling pathways in animal models. Based on existing research, C66 is emerging as a promising drug candidate for improving cardiovascular and renal health.

## Data Availability

Not applicable.

## List of abbreviations

3-NT: 3-nitrotyrosine
4-HNE: 4-hydroxynonenal
ACE: angiotensin-converting enzyme
AKT1: AKT serine/threonine kinase 1
Ang II: angiotensin II
C66: (2E,6E)-2,6-bis[(2-trifluoromethyl)benzylidene]cyclohexanone
CA: cinnamic aldehyde
CAV-1: caveolin 1
CBP: cyclic adenosine monophosphate response element binding protein
COX-2: cyclooxygenase 2
CTGF: connective tissue growth factor
CTGF: connective tissue growth factor
DN: diabetic nephropathy
EDG: electron donating groups
E_HOMO_: energy of the highest occupied molecular orbital
E_LUMO_: energy of the lowest unoccupied molecular orbital
ER: endoplasmic reticulum
ERK: extracellular signal-regulated kinase
EWG: electron withdrawing group
FN-1: fibronectin-1
FOXO3a: forkhead box O3a
HATs: histone acetyltransferases
HG: high glucose
HO1: heme oxygenase 1
HUVECs: human umbilical vein endothelial cells
ICAM-1: intercellular adhesion molecule 1
IFN-γ: interferon gamma
IkB: IkappaB kinase
IL-6: interleukin 6
IL-12: interleukin 12
IL-18: interleukin 18
IL-1β: interleukin 1 beta
IL-6: interleukin 6
IMPS: invalid metabolic panaceas
iNOS: inducible nitric oxide synthase
IRAK1: interleukin 1 receptor-associated kinase
JNK2: c-Jun N-terminal kinase 2
JNKi: JNK inhibitors
Keap1: Kelch-like ECH-associated protein 1
M6PRBP1: mannose-6-phosphate receptor binding protein 1
MACs: monocarbonyl analogs of curcumin
MAPK: mitogen-activated protein kinase
MCP-1: monocyte chemoattractant protein-1
MT: metallothioneins
NEDD4: neural precursor cell expressed developmentally down-regulated protein 4
NF-κB: nuclear factor kappa B
NO: nitric oxide
NQO1: NAD(P)H quinone dehydrogenase 1
Nrf2: nuclear factor erythroid 2–related factor 2
PAI-1: plasminogen activator inhibitor-1
PAINS: pan-assay interference compounds
PDCD 4: programmed cell death protein 4
PI3K: phosphoinositide 3-kinase
PPAR-γ: peroxisome proliferator- activated receptor gamma
RAS: renin-angiotensin system
ROS: reactive oxygen species
SFN: sulforaphane
SMAD: suppressor of mothers against decapentaplegic
SOD1: superoxide dismutase type 1
STZ: streptozotocin
TGF-β: transforming growth factor beta
TLR: toll-like receptor
TNF-α: tumor necrosis factor alpha
VCAM-1: vascular cell adhesion molecule 1

## References

[CR1] Ai J, Nie J, He J, Guo Q, Li M, Lei Y, Liu Y, Zhou Z, Zhu F, Liang M, Cheng Y, Hou FF (2015). GQ5 Hinders Renal Fibrosis in Obstructive Nephropathy by Selectively Inhibiting TGF-β-Induced Smad3 Phosphorylation. J Am Soc Nephrol.

[CR2] Al-Rifai N, Rücker H, Amslinger S (2013). Opening or Closing the Lock? When Reactivity Is the Key to Biological Activity. Chemistry.

[CR3] Altobelli E, Angeletti PM, Marziliano C, Mastrodomenico M, Giuliani AR, Petrocelli R. Potential therapeutic effects of curcumin on glycemic and lipid profile in uncomplicated type 2 diabetes-A meta-analysis of randomized controlled trial. Nutrients 2021, 13 (2). 10.3390/nu13020404. 10.3390/nu13020404 PMC7912109 33514002

[CR4] Amslinger S (2010). The Tunable Functionality of Alpha,Beta-Unsaturated Carbonyl Compounds Enables Their Differential Application in Biological Systems. ChemMedChem.

[CR5] Amslinger S, Al-Rifai N, Winter K, Wörmann K, Scholz R, Baumeister P, Wild M (2013). Reactivity Assessment of Chalcones by a Kinetic Thiol Assay. Org Biomol Chem.

[CR6] Anand P, Thomas SG, Kunnumakkara AB, Sundaram C, Harikumar KB, Sung B, Tharakan ST, Misra K, Priyadarsini IK, Rajasekharan KN, Aggarwal BB (2008). Biological Activities of Curcumin and Its Analogues (Congeners) Made by Man and Mother Nature. Biochem Pharmacol.

[CR7] Arshad L, Haque MA, Abbas Bukhari SN, Jantan I (2017). An Overview of Structure-Activity Relationship Studies of Curcumin Analogs as Antioxidant and Anti-Inflammatory Agents. Future Med Chem.

[CR8] Balogun E, Hoque M, Gong P, Killeen E, Green CJ, Foresti R, Alam J, Motterlini R (2003). Curcumin Activates the Haem Oxygenase-1 Gene via Regulation of Nrf2 and the Antioxidant-Responsive Element. Biochem J.

[CR9] Bennett BL, Sasaki DT, Murray BW, O’Leary EC, Sakata ST, Xu W, Leisten JC, Motiwala A, Pierce S, Satoh Y, Bhagwat SS, Manning AM, Anderson, D W. SP600125, an anthrapyrazolone inhibitor of jun N-terminal kinase. *Proc. Natl. Acad. Sci. U. S. A* 2001, *98* (24), 13681–13686. 10.1073/pnas.251194298. 10.1073/pnas.251194298 PMC61101 11717429

[CR10] Bitomsky N, Böhm M, Klempnauer K-H (2004). Transformation Suppressor Protein Pdcd4 Interferes with JNK-Mediated Phosphorylation of c-Jun and Recruitment of the Coactivator P300 by c-Jun. Oncogene.

[CR11] Burillo J, Marqués P, Jiménez B, González-Blanco C, Benito M, Guillén C. Insulin resistance and diabetes mellitus in Alzheimer’s disease. Cells 2021, 10 (5). 10.3390/cells10051236. 10.3390/cells10051236 PMC8157600 34069890

[CR12] Cai L, Wang J, Li Y, Sun X, Wang L, Zhou Z, Kang YJ (2005). Inhibition of Superoxide Generation and Associated Nitrosative Damage Is Involved in Metallothionein Prevention of Diabetic Cardiomyopathy. Diabetes.

[CR13] Cai L, Wang Y, Zhou G, Chen T, Song Y, Li X, Kang YJ (2006). Attenuation by Metallothionein of Early Cardiac Cell Death via Suppression of Mitochondrial Oxidative Stress Results in a Prevention of Diabetic Cardiomyopathy. J Am Coll Cardiol.

[CR14] Chen HY, Huang XR, Wang W, Li JH, Heuchel RL, Chung ACK, Lan HY (2011). The Protective Role of Smad7 in Diabetic Kidney Disease: Mechanism and Therapeutic Potential. Diabetes.

[CR15] Cho NH, Shaw JE, Karuranga S, Huang Y, da Rocha Fernandes JD, Ohlrogge AW, Malanda BIDF (2018). Diabetes Atlas: Global Estimates of Diabetes Prevalence for 2017 and Projections for 2045. Diabetes Res Clin Pract.

[CR16] Chung ACK, Zhang H, Kong Y-Z, Tan J-J, Huang XR, Kopp JB, Lan HY (2010). Advanced Glycation End-Products Induce Tubular CTGF via TGF-Beta-Independent Smad3 Signaling. J Am Soc Nephrol.

[CR17] Das U, Gul HI, Alcorn J, Shrivastav A, George T, Sharma RK, Nienaber KH, De Clercq E, Balzarini J, Kawase M, Kan N, Tanaka T, Tani S, Werbovetz KA, Yakovich AJ, Manavathu EK, Stables JP, Dimmock JR (2006). Cytotoxic 5-Aryl-1-(4-Nitrophenyl)-3-Oxo-1,4-Pentadienes Mounted on Alicyclic Scaffolds. Eur J Med Chem.

[CR18] Das U, Doroudi A, Das S, Bandy B, Balzarini J, De Clercq E, Dimmock JR (2008). E,E-2-Benzylidene-6-(Nitrobenzylidene)Cyclohexanones: Syntheses, Cytotoxicity and an Examination of Some of Their Electronic, Steric, and Hydrophobic Properties. Bioorg Med Chem.

[CR19] Das U, Sharma RK, Dimmock JR (2009). 1,5-Diaryl-3-Oxo-1,4-Pentadienes: A Case for Antineoplastics with Multiple Targets. Curr Med Chem.

[CR20] Day RM, Yang Y, Suzuki YJ, Stevens J, Pathi R, Perlmutter A, Fanburg BL, Lanzillo JJ (2001). Bleomycin Upregulates Gene Expression of Angiotensin-Converting Enzyme via Mitogen-Activated Protein Kinase and Early Growth Response 1 Transcription Factor. Am J Respir Cell Mol Biol.

[CR21] de Montellano PRO, de Montellano PRO (2005). Cytochrome P450: Structure, Mechanism, and Biochemistry, 3rd Ed.

[CR22] Dey N, Das F, Mariappan MM, Mandal CC, Ghosh-Choudhury N, Kasinath BS, Choudhury GG (2011). MicroRNA-21 Orchestrates High Glucose-Induced Signals to TOR Complex 1, Resulting in Renal Cell Pathology in Diabetes. J Biol Chem.

[CR23] Dimmock J, Sidhu K, Chen M, Reid R, Allen T, Kao G, Truitt G (1993). Evaluation of Some Mannich Bases of Cycloalkanones and Related Compounds for Cytotoxic Activity. Eur J Med Chem.

[CR24] Dimmock JR, Kandepu NM, Nazarali AJ, Kowalchuk TP, Motaganahalli N, Quail JW, Mykytiuk PA, Audette GF, Prasad L, Perjési P, Allen TM, Santos CL, Szydlowski J, De Clercq E, Balzarini J (1999). Conformational and Quantitative Structure-Activity Relationship Study of Cytotoxic 2-Arylidenebenzocycloalkanones. J Med Chem.

[CR25] Dimmock JR, Padmanilayam MP, Zello GA, Nienaber KH, Allen TM, Santos CL, De Clercq E, Balzarini J, Manavathu EK, Stables JP (2003). Cytotoxic Analogues of 2,6-Bis(Arylidene)Cyclohexanones. Eur J Med Chem.

[CR26] Eades G, Yang M, Yao Y, Zhang Y, Zhou Q (2011). MiR-200a Regulates Nrf2 Activation by Targeting Keap1 MRNA in Breast Cancer Cells. J Biol Chem.

[CR27] Ellison DH (2014). Bardoxolone Methyl in Type 2 Diabetes and Advanced Chronic Kidney Disease. N Engl J Med.

[CR28] Esatbeyoglu T, Huebbe P, Ernst IMA, Chin D, Wagner AE, Rimbach G (2012). Curcumin–from Molecule to Biological Function. Angew Chem Int Ed Engl.

[CR29] Eyries M, Agrapart M, Alonso A, Soubrier F (2002). Phorbol Ester Induction of Angiotensin-Converting Enzyme Transcription Is Mediated by Egr-1 and AP-1 in Human Endothelial Cells via ERK1/2 Pathway. Circ Res.

[CR30] Fan J, Li X, Yan Y-W, Tian X-H, Hou W-J, Tong H, Bai S-L (2012). Curcumin Attenuates Rat Thoracic Aortic Aneurysm Formation by Inhibition of the C-Jun N-Terminal Kinase Pathway and Apoptosis. Nutrition.

[CR31] Filler R, Kobayashu Y, Yagupolskii ML (1993). Organofluorine Compounds in Medicinal Chemistry and Biomedical Applications.

[CR32] Fiorentino TV, Prioletta A, Zuo P, Folli F (2013). Hyperglycemia-Induced Oxidative Stress and Its Role in Diabetes Mellitus Related Cardiovascular Diseases. Curr Pharm Des.

[CR33] Fiorillo C, Becatti M, Pensalfini A, Cecchi C, Lanzilao L, Donzelli G, Nassi N, Giannini L, Borchi E, Nassi P (2008). Curcumin Protects Cardiac Cells against Ischemia-Reperfusion Injury: Effects on Oxidative Stress, NF-KappaB, and JNK Pathways. Free Radic Biol Med.

[CR34] Gold R, Kappos L, Arnold DL, Bar-Or A, Giovannoni G, Selmaj K, Tornatore C, Sweetser MT, Yang M, Sheikh SI, Dawson KT (2012). DEFINE Study Investigators. Placebo-Controlled Phase 3 Study of Oral BG-12 for Relapsing Multiple Sclerosis. N Engl J Med.

[CR35] Gordon ON, Luis PB, Sintim HO, Schneider C (2015). Unraveling Curcumin Degradation: Autoxidation Proceeds through Spiroepoxide and Vinylether Intermediates En Route to the Main Bicyclopentadione. J Biol Chem.

[CR36] Gordon ON, Luis PB, Ashley RE, Osheroff N, Schneider C (2015). Oxidative Transformation of Demethoxy- and Bisdemethoxycurcumin: Products, Mechanism of Formation, and Poisoning of Human Topoisomerase IIα. Chem Res Toxicol.

[CR37] Gupta SC, Patchva S, Aggarwal BB (2013). Therapeutic Roles of Curcumin: Lessons Learned from Clinical Trials. AAPS J.

[CR38] Gupta SC, Kismali G, Aggarwal BB (2013). Curcumin, a Component of Turmeric: From Farm to Pharmacy. BioFactors.

[CR39] Hadzi-Petrushev N, Bogdanov J, Krajoska J, Ilievska J, Bogdanova-Popov B, Gjorgievska E, Mitrokhin V, Sopi R, Gagov H, Kamkin A, Mladenov M (2018). Comparative Study of the Antioxidant Properties of Monocarbonyl Curcumin Analogues C66 and B2BrBC in Isoproteranol Induced Cardiac Damage. Life Sci.

[CR40] Hadzi-Petrushev N, Angelovski M, Rebok K, Mitrokhin V, Kamkin A, Mladenov M (2019). Antioxidant and Anti-Inflammatory Effects of the Monocarbonyl Curcumin Analogs B2BRBC and C66 in Monocrotaline-Induced Right Ventricular Hypertrophy. J Biochem Mol Toxicol.

[CR41] Hagmann WK (2008). The Many Roles for Fluorine in Medicinal Chemistry. J Med Chem.

[CR42] Hajavi J, Momtazi AA, Johnston TP, Banach M, Majeed M, Sahebkar A, Curcumin (2017). A Naturally Occurring Modulator of Adipokines in Diabetes. J Cell Biochem.

[CR43] Hall ET, Bhalla V (2014). Is There a Sweet Spot for Nrf2 Activation in the Treatment of Diabetic Kidney Disease?. Diabetes.

[CR44] Hartner A, Cordasic N, Klanke B, Veelken R, Hilgers KF (2003). Strain Differences in the Development of Hypertension and Glomerular Lesions Induced by Deoxycorticosterone Acetate Salt in Mice. Nephrol Dial Transplant.

[CR45] Hayden MS, Ghosh S (2004). Signaling to NF-KappaB. Genes Dev.

[CR46] Hirata K, Shikata K, Matsuda M, Akiyama K, Sugimoto H, Kushiro M, Makino H (1998). Increased Expression of Selectins in Kidneys of Patients with Diabetic Nephropathy. Diabetologia.

[CR47] Huang J, Fu J, Liu B, Wang R, You TA, Synthetic curcuminoid, analog. (2E,6E)-2,6-Bis(2-(Trifluoromethyl)Benzylidene)Cyclohexanone, ameliorates impaired wound healing in streptozotocin-induced diabetic mice by increasing MiR-146a. Molecules 2020, 25 (4). 10.3390/molecules25040920. 10.3390/molecules25040920 PMC7070912 32092902

[CR48] Ishida T, Haneda M, Maeda S, Koya D, Kikkawa R (1999). Stretch-Induced Overproduction of Fibronectin in Mesangial Cells Is Mediated by the Activation of Mitogen-Activated Protein Kinase. Diabetes.

[CR49] Jiao Z, Chen J, Liu Y, Liu T, Chen K, Li G (2015). Role of ERK1/2 and JNK Phosphorylation in Iodine Contrast Agent-Induced Apoptosis in Diabetic Rat Kidneys. Ren Fail.

[CR50] Kang ES, Woo IS, Kim HJ, Eun SY, Paek KS, Kim HJ, Chang KC, Lee JH, Lee HT, Kim J-H, Nishinaka T, Yabe-Nishimura C, Seo HG (2007). Up-Regulation of Aldose Reductase Expression Mediated by Phosphatidylinositol 3-Kinase/Akt and Nrf2 Is Involved in the Protective Effect of Curcumin against Oxidative Damage. Free Radic Biol Med.

[CR51] Karelson M, Lobanov VS, Katritzky AR (1996). Quantum-Chemical Descriptors in QSAR/QSPR Studies. Chem Rev.

[CR52] Karukurichi KR, Wang L, Uzasci L, Manlandro CM, Wang Q, Cole PA (2010). Analysis of P300/CBP Histone Acetyltransferase Regulation Using Circular Permutation and Semisynthesis. J Am Chem Soc.

[CR53] Kato S, Luyckx VA, Ots M, Lee KW, Ziai F, Troy JL, Brenner BM, MacKenzie HS (1999). Renin-Angiotensin Blockade Lowers MCP-1 Expression in Diabetic Rats. Kidney Int.

[CR54] Kelly DJ, Cox AJ, Tolcos M, Cooper ME, Wilkinson-Berka JL, Gilbert RE (2002). Attenuation of Tubular Apoptosis by Blockade of the Renin-Angiotensin System in Diabetic Ren-2 Rats. Kidney Int.

[CR55] Kensler TW, Wakabayashi N, Biswal S (2007). Cell Survival Responses to Environmental Stresses via the Keap1-Nrf2-ARE Pathway. Annu Rev Pharmacol Toxicol.

[CR56] Keshari KR, Wilson DM, Sai V, Bok R, Jen K-Y, Larson P, Van Criekinge M, Kurhanewicz J, Wang ZJ (2015). Noninvasive in Vivo Imaging of Diabetes-Induced Renal Oxidative Stress and Response to Therapy Using Hyperpolarized 13 C Dehydroascorbate Magnetic Resonance. Diabetes.

[CR57] Kosugi T, Nakayama T, Heinig M, Zhang L, Yuzawa Y, Sanchez-Lozada LG, Roncal C, Johnson RJ, Nakagawa T (2009). Effect of Lowering Uric Acid on Renal Disease in the Type 2 Diabetic Db/Db Mice. Am J Physiol Renal Physiol.

[CR58] Kumar B, Singh V, Shankar R, Kumar K, Rawal RK (2017). Synthetic and Medicinal Prospective of Structurally Modified Curcumins. Curr Top Med Chem.

[CR59] Lam EW-F, Francis RE, Petkovic MFOXO (2006). Transcription Factors: Key Regulators of Cell Fate. Biochem Soc Trans.

[CR60] Lau ATY, Zhang J, Chiu J-F (2006). Acquired Tolerance in Cadmium-Adapted Lung Epithelial Cells: Roles of the c-Jun N-Terminal Kinase Signaling Pathway and Basal Level of Metallothionein. Toxicol Appl Pharmacol.

[CR61] Lee FTH, Cao Z, Long DM, Panagiotopoulos S, Jerums G, Cooper ME, Forbes JM (2004). Interactions between Angiotensin II and NF-KappaB-Dependent Pathways in Modulating Macrophage Infiltration in Experimental Diabetic Nephropathy. J Am Soc Nephrol.

[CR62] Li B, Cui W, Tan Y, Luo P, Chen Q, Zhang C, Qu W, Miao L, Cai L (2014). Zinc Is Essential for the Transcription Function of Nrf2 in Human Renal Tubule Cells in Vitro and Mouse Kidney in Vivo under the Diabetic Condition. J Cell Mol Med.

[CR63] Li C, Miao X, Wang S, Adhikari BK, Wang X, Sun J, Liu Q, Tong Q, Wang Y Novel Curcumin C66 that protects diabetes-induced aortic damage was associated with suppressing JNK2 and upregulating Nrf2 expression and function. Oxid. Med. Cell. Longev 2018a, 2018a, 5783239. https://doi.org/10.1155/2018a/5783239. 10.1155/2018/5783239 PMC6304198 30622669

[CR64] Li X, Xie X, Lian W, Shi R, Han S, Zhang H, Lu L, Li M (2018). Exosomes from Adipose-Derived Stem Cells Overexpressing Nrf2 Accelerate Cutaneous Wound Healing by Promoting Vascularization in a Diabetic Foot Ulcer Rat Model. Exp Mol Med.

[CR65] Li C, Miao X, Lou Y, Lu Z, Adhikari BK, Wang Y, Liu Q, Sun J, Wang Y (2018). Cardioprotective Effects of the Novel Curcumin Analogue C66 in Diabetic Mice Is Dependent on JNK2 Inactivation. J Cell Mol Med.

[CR66] Liang G, Yang S, Jiang L, Zhao Y, Shao L, Xiao J, Ye F, Li Y, Li X (2008). Synthesis and Anti-Bacterial Properties of Mono-Carbonyl Analogues of Curcumin. Chem Pharm Bull (Tokyo).

[CR67] Liang G, Shao L, Wang Y, Zhao C, Chu Y, Xiao J, Zhao Y, Li X, Yang S (2009). Exploration and Synthesis of Curcumin Analogues with Improved Structural Stability Both in Vitro and in Vivo as Cytotoxic Agents. Bioorg Med Chem.

[CR68] Liu Y, Wang Y, Miao X, Zhou S, Tan Y, Liang G, Zheng Y, Liu Q, Sun J, Cai L (2014). Inhibition of JNK by Compound C66 Prevents Pathological Changes of the Aorta in STZ-Induced Diabetes. J Cell Mol Med.

[CR69] Maheshwari RK, Singh AK, Gaddipati J, Srimal RC (2006). Multiple Biological Activities of Curcumin: A Short Review. Life Sci.

[CR70] Maritim AC, Sanders RA, Watkins JB, Diabetes (2003). Oxidative Stress, and Antioxidants: A Review. J Biochem Mol Toxicol.

[CR71] Marton LT, Pescinini-E-Salzedas LM, Camargo MEC, Barbalho SM, Haber JFDS, Sinatora RV, Detregiachi CRP, Girio RJS, Buchaim DV (2021). Cincotto Dos Santos Bueno, P. The Effects of Curcumin on Diabetes Mellitus: A Systematic Review. Front Endocrinol (Lausanne).

[CR72] Miao X, Wang Y, Sun J, Sun W, Tan Y, Cai L, Zheng Y, Su G, Liu Q, Wang Y (2013). Zinc Protects against Diabetes-Induced Pathogenic Changes in the Aorta: Roles of Metallothionein and Nuclear Factor (Erythroid-Derived 2)-like 2. Cardiovasc Diabetol.

[CR73] Müller K, Faeh C, Diederich F (2007). Fluorine in Pharmaceuticals: Looking beyond Intuition. Science.

[CR74] Nagib DA, MacMillan DWC (2011). Trifluoromethylation of Arenes and Heteroarenes by Means of Photoredox Catalysis. Nature.

[CR75] Nakamura R, Egashira K, Arimura K, Machida Y, Ide T, Tsutsui H, Shimokawa H, Takeshita A (2001). Increased Inactivation of Nitric Oxide Is Involved in Impaired Coronary Flow Reserve in Heart Failure. Am J Physiol Heart Circ Physiol.

[CR76] Navarro-González JF, Mora-Fernández C, de MurosFuentes M, García-Pérez J (2011). Inflammatory Molecules and Pathways in the Pathogenesis of Diabetic Nephropathy. Nat Rev Nephrol.

[CR77] Nelson KM, Dahlin JL, Bisson J, Graham J, Pauli GF, Walters MA (2017). The Essential Medicinal Chemistry of Curcumin. J Med Chem.

[CR78] Nepali K, Lee H-Y, Liou J-P (2019). Nitro-Group-Containing Drugs. J Med Chem.

[CR79] Noonan WT, Banks RO renal function and glucose transport in male and female mice with diet-induced type II diabetes mellitus. *Proc. Soc. Exp. Biol. Med* 2000, *225* (3), 221–230. 10.1046/j.1525-1373.2000.22528.x. 10.1046/j.1525-1373.2000.22528.x 11082217

[CR80] Pan Y, Wang Y, Cai L, Cai Y, Hu J, Yu C, Li J, Feng Z, Yang S, Li X, Liang G (2012). Inhibition of High Glucose-Induced Inflammatory Response and Macrophage Infiltration by a Novel Curcumin Derivative Prevents Renal Injury in Diabetic Rats. Br J Pharmacol.

[CR81] Pan Y, Zhang X, Wang Y, Cai L, Ren L, Tang L, Wang J, Zhao Y, Wang Y, Liu Q, Li X, Liang G (2013). Targeting JNK by a New Curcumin Analog to Inhibit NF-KB-Mediated Expression of Cell Adhesion Molecules Attenuates Renal Macrophage Infiltration and Injury in Diabetic Mice. PLoS ONE.

[CR82] Pan Y, Huang Y, Wang Z, Fang Q, Sun Y, Tong C, Peng K, Wang Y, Miao L, Cai L, Zhao Y, Liang G (2014). Inhibition of MAPK-Mediated ACE Expression by Compound C66 Prevents STZ-Induced Diabetic Nephropathy. J Cell Mol Med.

[CR83] Panahi Y, Khalili N, Sahebi E, Namazi S, Karimian MS, Majeed M, Sahebkar A (2017). Antioxidant Effects of Curcuminoids in Patients with Type 2 Diabetes Mellitus: A Randomized Controlled Trial. Inflammopharmacology.

[CR84] Panahi Y, Khalili N, Sahebi E, Namazi S, Reiner Ž, Majeed M, Sahebkar A (2017). Curcuminoids Modify Lipid Profile in Type 2 Diabetes Mellitus: A Randomized Controlled Trial. Complement Ther Med.

[CR85] Park CW, Kim JH, Lee JH, Kim YS, Ahn HJ, Shin YS, Kim SY, Choi EJ, Chang YS, Bang BK, Lee JW (2000). High Glucose-Induced Intercellular Adhesion Molecule-1 (ICAM-1) Expression through an Osmotic Effect in Rat Mesangial Cells Is PKC-NF-Kappa B-Dependent. Diabetologia.

[CR86] Parsamanesh N, Moossavi M, Bahrami A, Butler AE, Sahebkar A (2018). Therapeutic Potential of Curcumin in Diabetic Complications. Pharmacol Res.

[CR87] Peng Z, Peng L, Fan Y, Zandi E, Shertzer HG, Xia Y (2007). A Critical Role for IkappaB Kinase Beta in Metallothionein-1 Expression and Protection against Arsenic Toxicity. J Biol Chem.

[CR88] Pivari F, Mingione A, Brasacchio C, Soldati L. curcumin and type 2 diabetes mellitus: prevention and treatment. Nutrients 2019, 11 (8). 10.3390/nu11081837. 10.3390/nu11081837 PMC6723242 31398884

[CR89] Purser S, Moore PR, Swallow S, Gouverneur V (2008). Fluorine in Medicinal Chemistry. Chem Soc Rev.

[CR90] Qian Y, Zhong P, Liang D, Xu Z, Skibba M, Zeng C, Li X, Wei T, Wu L, Liang GA (2015). Newly Designed Curcumin Analog Y20 Mitigates Cardiac Injury via Anti-Inflammatory and Anti-Oxidant Actions in Obese Rats. PLoS ONE.

[CR91] Qu W, Fuquay R, Sakurai T, Waalkes MP (2006). Acquisition of Apoptotic Resistance in Cadmium-Induced Malignant Transformation: Specific Perturbation of JNK Signal Transduction Pathway and Associated Metallothionein Overexpression. Mol Carcinog.

[CR92] Rivera-Mancía S, Trujillo J, Chaverri JP (2018). Utility of Curcumin for the Treatment of Diabetes Mellitus: Evidence from Preclinical and Clinical Studies. J Nutr Intermed Metab.

[CR93] Schneider C, Gordon ON, Edwards RL, Luis PB (2015). Degradation of Curcumin: From Mechanism to Biological Implications. J Agric Food Chem.

[CR94] Senoner T, Dichtl W. Oxidative stress in cardiovascular diseases: still a therapeutic target? Nutrients 2019, 11 (9). 10.3390/nu11092090. 10.3390/nu11092090 PMC6769522 31487802

[CR95] Shakman KB, Mazziotti DA (2007). Assessing the Efficacy of Nonsteroidal Anti-Inflammatory Drugs through the Quantum Computation of Molecular Ionization Energies. J Phys Chem A.

[CR96] Shetty D, Kim YJ, Shim H, Snyder JP (2014). Eliminating the Heart from the Curcumin Molecule: Monocarbonyl Curcumin Mimics (MACs). Molecules.

[CR97] Sklenar H, Jäger J (1979). Molecular Structure-Biological Activity Relationships on the Basis of Quantum-Chemical Calculations. Int J Quantum Chem.

[CR98] Soetikno V, Sari FR, Lakshmanan AP, Arumugam S, Harima M, Suzuki K, Kawachi H, Watanabe K (2013). Curcumin Alleviates Oxidative Stress, Inflammation, and Renal Fibrosis in Remnant Kidney through the Nrf2-Keap1 Pathway. Mol Nutr Food Res.

[CR99] Soetikno V, Suzuki K, Veeraveedu PT, Arumugam S, Lakshmanan AP, Sone H, Watanabe K (2013). Molecular Understanding of Curcumin in Diabetic Nephropathy. Drug Discov Today.

[CR100] Song Y, Wang J, Li Y, Du Y, Arteel GE, Saari JT, Kang YJ, Cai L (2005). Cardiac Metallothionein Synthesis in Streptozotocin-Induced Diabetic Mice, and Its Protection against Diabetes-Induced Cardiac Injury. Am J Pathol.

[CR101] Stamenkovska M, Thaçi Q, Hadzi-Petrushev N, Angelovski M, Bogdanov J, Reçica S, Kryeziu I, Gagov H, Mitrokhin V, Kamkin A, Schubert R, Mladenov M, Sopi RB (2020). Curcumin Analogs (B2BrBC and C66) Supplementation Attenuates Airway Hyperreactivity and Promote Airway Relaxation in Neonatal Rats Exposed to Hyperoxia. Physiol Rep.

[CR102] Stamenkovska M, Hadzi-Petrushev N, Nikodinovski A, Gagov H, Atanasova-Panchevska N, Mitrokhin V, Kamkin A, Mladenov M (2021). Application of Curcumine and Its Derivatives in the Treatment of Cardiovascular Diseases: A Review. Int J Food Prop.

[CR103] Sun A, Lu YJ, Hu H, Shoji M, Liotta DC, Snyder JP (2009). Curcumin Analog Cytotoxicity against Breast Cancer Cells: Exploitation of a Redox-Dependent Mechanism. Bioorg Med Chem Lett.

[CR104] Sun X, Liu Y, Li C, Wang X, Zhu R, Liu C, Liu H, Wang L, Ma R, Fu M, Zhang D, Li Y Recent advances of curcumin in the prevention and treatment of renal fibrosis. *Biomed Res. Int* 2017, *2017*, 2418671. 10.1155/2017/2418671. 10.1155/2017/2418671 PMC5435901 28546962

[CR105] Sunters A, Madureira PA, Pomeranz KM, Aubert M, Brosens JJ, Cook SJ, Burgering BMT, Coombes RC, Lam E (2006). W.-F. Paclitaxel-Induced Nuclear Translocation of FOXO3a in Breast Cancer Cells Is Mediated by c-Jun NH2-Terminal Kinase and Akt. Cancer Res.

[CR106] Tesch GH, Macrophages, Nephropathy D (2010). Semin Nephrol.

[CR107] Tsai S-Y, Huang Y-L, Yang W-H, Tang C-H (2012). Hepatocyte Growth Factor-Induced BMP-2 Expression Is Mediated by c-Met Receptor, FAK, JNK, Runx2, and P300 Pathways in Human Osteoblasts. Int Immunopharmacol.

[CR108] Uruno A, Furusawa Y, Yagishita Y, Fukutomi T, Muramatsu H, Negishi T, Sugawara A, Kensler TW, Yamamoto M (2013). The Keap1-Nrf2 System Prevents Onset of Diabetes Mellitus. Mol Cell Biol.

[CR109] Velic A, Laturnus D, Chhoun J, Zheng S, Epstein P, Carlson E (2013). Diabetic Basement Membrane Thickening Does Not Occur in Myocardial Capillaries of Transgenic Mice When Metallothionein Is Overexpressed in Cardiac Myocytes. Anat Rec (Hoboken).

[CR110] Villeneuve LM, Reddy MA, Natarajan R, Epigenetics (2011). Deciphering Its Role in Diabetes and Its Chronic Complications. Clin Exp Pharmacol Physiol.

[CR111] Wang Y, Rangan GK, Goodwin B, Tay YC, Harris DC, Lipopolysaccharide-Induced (2000). MCP-1 Gene Expression in Rat Tubular Epithelial Cells Is Nuclear Factor-KappaB Dependent. Kidney Int.

[CR112] Wang Y, Zhang Z, Sun W, Tan Y, Liu Y, Zheng Y, Liu Q, Cai L, Sun J Sulforaphane attenuation of type 2 diabetes-induced aortic damage was associated with the upregulation of Nrf2 expression and function. Oxid. Med. Cell. Longev 2014a, 2014a, 123963. https://doi.org/10.1155/2014a/123963. 10.1155/2014/123963 PMC3953421 24707343

[CR113] Wang Y, Zhou S, Sun W, McClung K, Pan Y, Liang G, Tan Y, Zhao Y, Liu Q, Sun J, Cai L (2014). Inhibition of JNK by Novel Curcumin Analog C66 Prevents Diabetic Cardiomyopathy with a Preservation of Cardiac Metallothionein Expression. Am J Physiol Endocrinol Metab.

[CR114] Wang J-Y, Gao Y-B, Zhang N, Zou D-W, Wang P, Zhu Z-Y, Li J-Y, Zhou S-N, Wang S-C, Wang Y-Y, Yang J-K (2014). MiR-21 Overexpression Enhances TGF-Β1-Induced Epithelial-to-Mesenchymal Transition by Target Smad7 and Aggravates Renal Damage in Diabetic Nephropathy. Mol Cell Endocrinol.

[CR115] Wang Y, Wang Y, Luo M, Wu H, Kong L, Xin Y, Cui W, Zhao Y, Wang J, Liang G, Miao L, Cai L (2015). Novel Curcumin Analog C66 Prevents Diabetic Nephropathy via JNK Pathway with the Involvement of P300/CBP-Mediated Histone Acetylation. Biochim Biophys Acta.

[CR116] Wang R-Y, Liu L-H, Liu H, Wu K-F, An J, Wang Q, Liu Y, Bai L-J, Qi B-M, Qi B-L, Zhang L (2018). Nrf2 Protects against Diabetic Dysfunction of Endothelial Progenitor Cells via Regulating Cell Senescence. Int J Mol Med.

[CR117] Wen Y, Gu J, Li S-L, Reddy MA, Natarajan R, Nadler JL (2006). Elevated Glucose and Diabetes Promote Interleukin-12 Cytokine Gene Expression in Mouse Macrophages. Endocrinology.

[CR118] Weseler AR, Bast A (2010). Oxidative Stress and Vascular Function: Implications for Pharmacologic Treatments. Curr Hypertens Rep.

[CR119] Wu H, Kong L, Tan Y, Epstein PN, Zeng J, Gu J, Liang G, Kong M, Chen X, Miao L, Cai L (2016). C66 Ameliorates Diabetic Nephropathy in Mice by Both Upregulating NRF2 Function via Increase in MiR-200a and Inhibiting MiR-21. Diabetologia.

[CR120] Xu J, Wang G, Wang Y, Liu Q, Xu W, Tan Y, Cai L (2009). Diabetes- and Angiotensin II-Induced Cardiac Endoplasmic Reticulum Stress and Cell Death: Metallothionein Protection. J Cell Mol Med.

[CR121] Yang Y, Wang J, Qin L, Shou Z, Zhao J, Wang H, Chen Y, Chen J (2007). Rapamycin Prevents Early Steps of the Development of Diabetic Nephropathy in Rats. Am J Nephrol.

[CR122] Ye G, Metreveli NS, Ren J, Epstein PN (2003). Metallothionein Prevents Diabetes-Induced Deficits in Cardiomyocytes by Inhibiting Reactive Oxygen Species Production. Diabetes.

[CR123] Yonemoto S, Machiguchi T, Nomura K, Minakata T, Nanno M, Yoshida H (2006). Correlations of Tissue Macrophages and Cytoskeletal Protein Expression with Renal Fibrosis in Patients with Diabetes Mellitus. Clin Exp Nephrol.

[CR124] Zhang Y, Pan K-L, He F, Chen L-F, Liu Z-G, Liang G (2015). Crystal Structure of (2E,6E)-2,6-Bis[2-(Trifluoromethyl)Benzylidene]Cyclohexanone, C22H16F6O. Z für Krist - New Cryst Struct.

[CR125] Zhao C, Liu Z, Liang G (2013). Promising Curcumin-Based Drug Design: Mono-Carbonyl Analogues of Curcumin (MACs). Curr Pharm Des.

[CR126] Zheng H, Whitman SA, Wu W, Wondrak GT, Wong PK, Fang D, Zhang DD (2011). Therapeutic Potential of Nrf2 Activators in Streptozotocin-Induced Diabetic Nephropathy. Diabetes.

[CR127] Zheng Q-T, Yang Z-H, Yu L-Y, Ren Y-Y, Huang Q-X, Liu Q, Ma X-Y, Chen Z-K, Wang Z-B, Zheng X (2017). Synthesis and Antioxidant Activity of Curcumin Analogs. J Asian Nat Prod Res.

[CR128] Zhong X, Chung ACK, Chen HY, Dong Y, Meng XM, Li R, Yang W, Hou FF, Lan HY (2013). MiR-21 Is a Key Therapeutic Target for Renal Injury in a Mouse Model of Type 2 Diabetes. Diabetologia.

[CR129] Zhou L, Xue H, Wang Z, Ni J, Yao T, Huang Y, Yu C, Lu L (2012). Angiotensin-(1–7) Attenuates High Glucose-Induced Proximal Tubular Epithelial-to-Mesenchymal Transition via Inhibiting ERK1/2 and P38 Phosphorylation. Life Sci.

[CR130] Zhou J, Du X, Long M, Zhang Z, Zhou S, Zhou J, Qian G (2016). Neuroprotective Effect of Berberine Is Mediated by MAPK Signaling Pathway in Experimental Diabetic Neuropathy in Rats. Eur J Pharmacol.

[CR131] Zoete V, Rougée M, Dinkova-Kostova AT, Talalay P, Bensasson RV (2004). Redox Ranking of Inducers of a Cancer-Protective Enzyme via the Energy of Their Highest Occupied Molecular Orbital. Free Radic Biol Med.

